# ﻿Global phylogeny of the family *Gomphillaceae* (*Ascomycota*, *Graphidales*) sheds light on the origin, diversification and endemism in foliicolous lineages

**DOI:** 10.3897/imafungus.16.144194

**Published:** 2025-02-17

**Authors:** Elise Lebreton, Damien Ertz, Robert Lücking, Andre Aptroot, Fabian Carriconde, Claudine Ah-Peng, Jen-Pan Huang, Ko-Hsuan Chen, Pierre-Louis Stenger, Marcela Eugenia da Silva Cáceres, Pieter van den Boom, Emmanuël Sérusiaux, Nicolas Magain

**Affiliations:** 1 Biology, Evolution, Conservation, Inbios Research Center, University of Liège, Quartier Vallée 1, B-4000 Liège, Belgium University of Liège Liège Belgium; 2 Department of Research, Meise Botanic Garden, B-1860 Meise, Belgium Meise Botanic Garden Meise Belgium; 3 Service Général de l’Enseignement Supérieur et de la Recherche Scientifique, Fédération Wallonie-Bruxelles, B-1080 Bruxelles, Belgium Service Général de l’Enseignement Supérieur et de la Recherche Scientifique, Fédération Wallonie-Bruxelles Bruxelles Belgium; 4 Botanischer Garten und Botanisches Museum Berlin, Freie Universität Berlin, 14195 Berlin, Germany Freie Universität Berlin Berlin Germany; 5 Laboratório de Botânica / Liquenologia, Instituto de Biociências, Universidade Federal de Mato Grosso do Sul, CEP 79070-900, Campo Grande, Mato Grosso do Sul, Brazil Universidade Federal de Mato Grosso do Sul Campo Grande Brazil; 6 Institut Agronomique néo-Calédonien (IAC), Équipe « Sol & Végétation » (SolVeg), 98800 Nouméa, New Caledonia (Fr) Institut Agronomique néo-Calédonien (IAC), Équipe « Sol & Végétation » (SolVeg) Nouméa New Caledonia (Fr); 7 UMR PVBMT, Université de La Réunion, Saint-Pierre, France MR PVBMT, Université de La Réunion Saint-Pierre France; 8 OSU-R, Université de La Réunion, Saint-Denis, France OSU-R, Université de La Réunion Saint-Denis France; 9 Biodiversity Research Center, Academia Sinica, 11529, Taipei, Taiwan Biodiversity Research Center, Academia Sinica Taipei Taiwan; 10 Omicsphere Analytics, 19 rue Philippe Maupas, 37250 Montbazon, France Omicsphere Analytics Montbazon France; 11 Departamento de Biologia, Universidade Federal de Sergipe, CEP 49107-230, São Cristóvão, Sergipe, Brazil Universidade Federal de Sergipe São Cristóvão Brazil; 12 Arafura 16, 5691JA, Son, Netherlands Unaffiliated Son Netherlands

**Keywords:** Ancestral geographic ranges, endemism, lichens, Neotropics, Palaeotropics, species delimitation

## Abstract

Foliicolous lichens grow on living leaves of vascular plants. They are mostly found in tropical to subtropical or temperate rainforests. Many phenotype-based species are considered as pantropical or even sub-cosmopolitan, either attributed to old ages, having existed prior to continental breakups or long-distance dispersal. We built a much expanded, global phylogeny of *Gomphillaceae*, the most diverse group of leaf-dwelling lichenised fungi. Our sampling encompassed six major biodiversity hotspots: MIOI (Madagascar and the Indian Ocean Islands), the Caribbean, New Caledonia, the Colombian Chocó, Mesoamerica and the Atlantic coast of Brazil. It was based on multilocus sequence data (mtSSU rDNA, nuLSU rDNA and RPB1), including 2207 sequences of 1256 specimens. Species delimitation methods combined with a phenotype matrix identified 473 putative species. Amongst these, 104 are confirmed as described, 213 are classified as cryptic or near cryptic (hidden diversity), 100 represent new species to science (identified on the basis of phenotype) and 56 remain unidentified. Amongst the 104 species with a valid name, 40.5% are distributed across 2–5 continents (lichenogeographical regions) by applying the phenotype-based species concept. However, using the integrative approach to delineate species, this estimate is reduced to 9%. We estimate the global species richness of *Gomphillaceae* at 1,861–2,356 species. The timing of species-level divergences suggests that the current distribution of foliicolous lichens is shaped more by long-distance dispersal and rapid diversification than by vicariance. The origin of the family and major clades appears to be in the Neotropics, with subsequent numerous dispersal events. Our results support the separation of three major lineages, corresponding to the former families *Asterothyriaceae*, *Gomphillaceae* s.str. and *Solorinellaceae*, which should be recognised at the subfamily level.

## ﻿Introduction

### ﻿Lichens and paradigm shift

Lichen-forming fungi, following Index Fungorum (http://www.indexfungorum.org), make up around 12.5% of the 161,288 known fungal species (Jaklitsch et al. 2016; Lücking et al. 2017a) and are found in all terrestrial ecosystems (Purvis 2000; Lücking and Spribille 2024). While traditionally considered to have widespread distribution ranges, especially compared to vascular plants (Lücking 2003; Feuerer and Hawksworth 2007), recent studies have challenged the “everything small is everywhere” paradigm in lichens (Leavitt et al. 2018a; Ruprecht et al. 2020; Simon et al. 2022; Magain et al. 2023; Pérez-Vargas et al. 2024). Indeed, molecular data have significantly modified our understanding of lichen diversity and geographic ranges, questioning morphology-based species concepts (Grube and Kroken 2000; Crespo and Pérez-Ortega 2009; Crespo and Lumbsch 2010; Lumbsch and Leavitt 2011; Lücking et al. 2021). Species delimitation methods have uncovered cryptic diversity within morphologically similar lichens, suggesting that what appears to be a widespread species may actually consist of genetically distinct, regionally restricted lineages (Singh et al. 2015; Leavitt et al. 2016a; Magain et al. 2018; Mercado-Díaz et al. 2020; Dal-Forno et al. 2022). Hidden diversity has been shown in presumably known taxa, with ratios sometimes exceeding 10:1, such as in *Coraglabrata* (265:1) (Lücking et al. 2014, 2017b), *Lecanorapolytropa* (75:1) (Zhang et al. 2022), *Parmeliasaxatilis* (12:1) (Divakar et al. 2016; Molina et al. 2017; Crespo et al. 2020), *Pseudocyphellariacrocata* (25:1) (Lücking et al. 2017c) and *Stictafuliginosa* (20:1) (Di Meglio and Goward 2023). Consequently, the high levels of candidate species identified at local scales, with limited regional overlap, indicate a higher rate of endemism in lichens than previously assumed (Sérusiaux et al. 2011; Mercado-Díaz et al. 2014, 2023; Dal-Forno et al. 2017; Simon et al. 2018; Moncada et al. 2020; Blázquez et al. 2024; Masson et al. 2024). Nevertheless, some species confirmed through molecular tools still occupy extensive geographic ranges (Otálora et al. 2010; Geml et al. 2012; Amo de Paz et al. 2012; Fernández-Mendoza and Printzen 2013; Leavitt et al. 2013; Núñez-Zapata et al. 2015). Amongst distribution patterns in lichens, intercontinental disjunctions are common at the genus level, but have been presumed for many species as well (Galloway 2008; Werth 2011). Hypotheses explaining these patterns include vicariance through continental drift or fragmentation (Lücking 2003; Printzen et al. 2003; Walser et al. 2005) or, alternatively, more recent long-distance dispersal facilitated by the microscopic size of spores and asexual propagules (Buschbom 2007; Leavitt et al. 2013; Cubas et al. 2018; Leavitt et al. 2018a; Garrido-Benavent et al. 2021) or a combination of both (Lücking and Kalb 2001; Del-Prado et al. 2013; Garrido-Benavent et al. 2018). Consequently, recent studies on lichen biogeography tend to integrate molecular dating approaches to investigate the roles of dispersal and vicariance events in shaping global patterns of taxa distribution (Leavitt and Lumbsch 2016).

### ﻿Foliicolous lichens

Amongst lichens, foliicolous taxa are amongst the best-documented in terms of their taxonomy and distribution (Santesson 1952; Lücking 2008; Van Den Broeck et al. 2014; Wang et al. 2020; Van den Boom 2021; Miyazawa et al. 2022; Farkas 2023; Zhu et al. 2023). A biogeographic analysis of around 800 species, conducted by Lücking (2003) and based on Takhtajan’s floristic regions (Takhtajan 1986), identified six major lichenogeographical regions: (1) Neotropics, (2) African Palaeotropics, (3) Eastern Palaeotropics, (4) Valdivian region, (5) Tethyan region and (6) Neozealandic-Tasmanian region. According to this study, these regions shared 57–77% of the species, with 21% being cosmopolitan or pantropical, 19% disjunct across multiple continents and 60% restricted to a single tropical area. Subcosmopolitan, pantropical and otherwise intercontinental foliicolous lichen species have been regularly reported in inventories from various regions, such as the Valdivian Rainforest in southern Chile and Argentina (Lücking et al. 2003, 62–73% intercontinental, 31% pantropical), Mexico (Herrera-Campos et al. 2004, 63% intercontinental, 43% pantropical), Brazil (Cáceres et al. 2000, 66% intercontinental, 46% pantropical), Spain (Llop and Gómez-Bolea 2006, 50% intercontinental, 28% pantropical), New Caledonia (Lücking and Kalb 2001, 94% intercontinental, 64% pantropical), Ivory Coast (Lücking et al. 1998a, 86% intercontinental, 54% pantropical) and the Fiji Islands (Farkas 2023, 60% pantropical), suggesting low levels of endemism among these lichens. However, recent studies using molecular methods on foliicolous lichens have highlighted how morphology-based taxonomy may have underestimated species diversity at the regional scale. Hidden diversity was shown in foliicolous species of the genus *Strigula* in Asia, which were previously considered to have widespread distributions (Jayalal et al. 2013; Jiang et al. 2017a, b; Woo et al. 2020; Jiang et al. 2021), even when allowing a certain level of infraspecific genetic variability (Oh et al. 2019). In the Neotropics, strong genetic diversity within and between species and species complexes has been revealed in the genera *Porina* (Baloch and Grube 2006, 2009), *Gyalectidium* and *Tricharia* (Xavier-Leite et al. 2022).

The mechanisms behind the emergence of new lineages in foliicolous lichens remain largely unexplored. Given that rainwater serves as the primary dispersal vector for these lichens, through either running water or splash mechanisms (Sérusiaux 1995; Lücking 2001; Lücking and Bernecker-Lücking 2002; Sanders et al. 2016), it is expected that diaspores are typically dispersed over short distances (< 1 m). Indeed, studies comparing foliicolous lichens on Pacific islands (Cocos Island and New Caledonia) suggest that dispersal distances are limited and are unlikely to exceed 1500 km (Lücking and Lücking 1995; Lücking and Kalb 2001). Based on this, vicariance rather than long-distance dispersal was proposed to explain the similarities between foliicolous lichen communities between the African Palaeotropics and the Neotropics, for instance using the example of Chroodiscus (Graphidaceae) (Lücking et al. 1998a, 2008; Lücking 2001, 2003). This hypothesis would suggest a phylogenetic age of species shared between the Neotropics and the African Palaeotropics around 90–150 million years, prior to the breakup of Gondwana. However, recent molecular dating studies on the *Gomphillaceae* family (Xavier-Leite et al. 2022) have rejected this hypothesis, showing that foliicolous lineages of this family representing species complexes diversified long after the Cretaceous–Palaeogene boundary (K–Pg boundary), primarily during the Miocene (5–23 million years ago).

### ﻿*Gomphillaceae* as a case study

*Gomphillaceae* Walt. Watson offers valuable insights into various evolutionary phenomena in lichenised fungi due to its vast diversity in morpho-anatomical and ecogeographical traits (Lücking et al. 2005; Lücking 2008; Xavier-Leite et al. 2022). This family is the dominant element of foliicolous lichen communities in tropical and subtropical wet forests (Nowak and Winkler 1970, 1975; Rogers et al. 1994; Mateus et al. 2012; Flakus 2013; Santos et al. 2020; Farias et al. 2021) and is present in all major lichenogeographical regions defined by Lücking (2003), making it an excellent model for testing historical hypotheses on lichen biogeography. With around 459 lichenised and lichenicolous or fungicolous species known to date and the ongoing sequencing efforts (Miyazawa et al. 2022, 2023; Zhu et al. 2023; Lücking et al. 2024), this family has the best sequence coverage amongst foliicolous taxa. While most species are foliicolous, others are found on rocks (Vězda 1966), soil, mosses (Ferraro and Lücking 2005) and bark (Kalb and Vězda 1988), including some specifically on twigs of shrubs (Sérusiaux 1998). A few species are lichenicolous (Lücking and Sérusiaux 1992; Lücking 1997a; Lücking and Kalb 2002; Suija et al. 2018; Flakus et al. 2019) or mycoparasitic (Guterres et al. 2020).

The systematics of this family have been historically controversial, as in some concepts, it results from the fusion of *Gomphillaceae* with one or two families (*Asterothyriaceae* and *Solorinellaceae*) (Baloch et al. 2010; Rivas Plata et al. 2012; Lücking et al. 2017a; Xavier-Leite et al. 2022). As the relationships amongst these former families were not supported in the most recent phylogeny, Xavier-Leite et al. (2022) defended a broad definition of *Gomphillaceae*, encompassing the former families *Asterothyriaceae* and *Solorinellaceae*.

### ﻿Study objectives

The main goals of this study are to assess species diversity and reconstruct the biogeographical history of family *Gomphillaceae* at a global scale. Specifically, we aim to answer the following questions: 1) Are pantropical species common or do most species have more restricted distributions? 2) What is the estimated species richness in family *Gomphillaceae*? 3) What is the biogeographical history of the family in terms of vicariance and long-term dispersal?

Starting with the existing dataset on foliicolous *Gomphillaceae* from the Neotropics (Xavier-Leite et al. 2022), this study aims to expand the phylogeny and increase the backbone resolution, including relationships with the former families *Asterothyriaceae* and *Solorinellaceae* by incorporating 913 specimens collected from territories encompassing six major tropical biodiversity hotspots worldwide.

## ﻿Materials and methods

### ﻿Sampling

**Figure 1.**
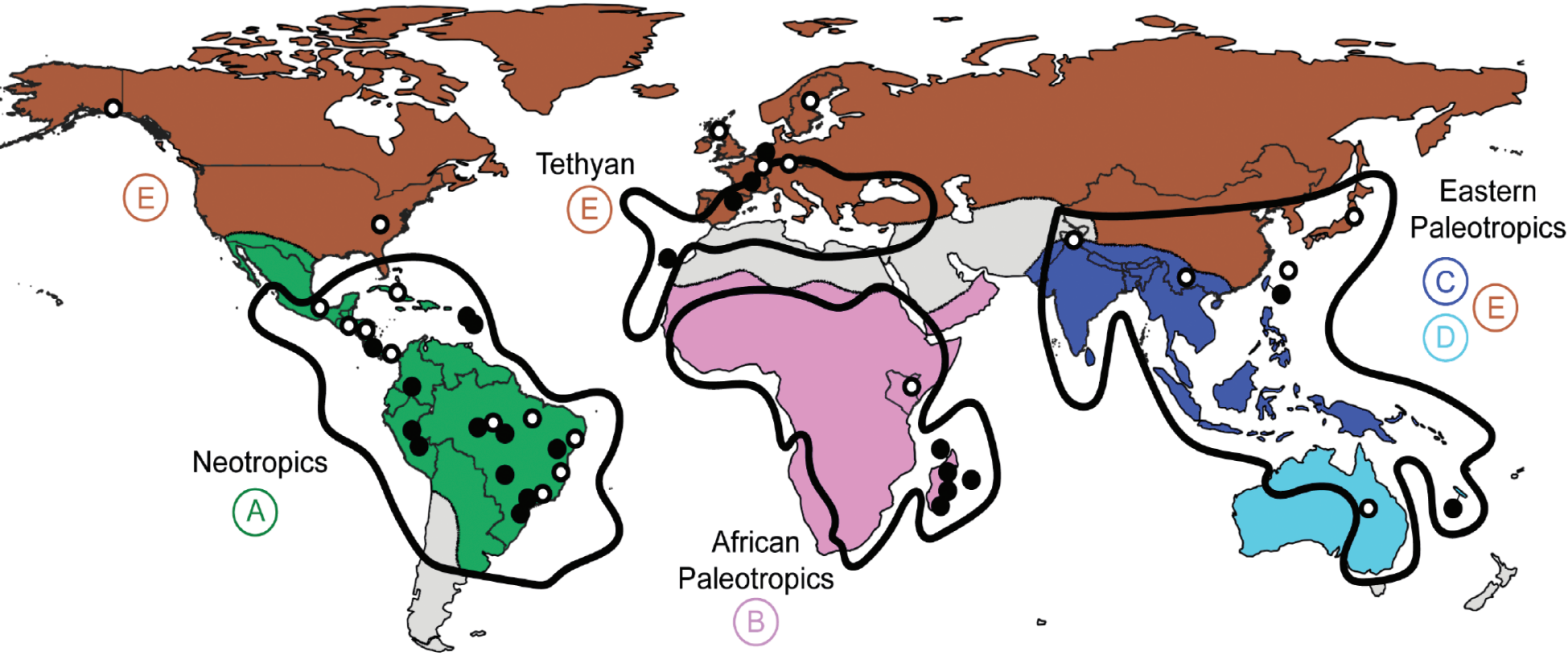
World map illustrating the geographic origins of DNA-confirmed samples from collections of *Gomphillaceae*. Black dots represent new collections investigated by the authors and white dots collections previously studied by other authors. The black lines outline the distribution within the four lichenogeographical regions as defined by Lücking (2003). Coloured areas represent the distribution within the five of the eight floristic realms according to Liu et al. (2023): A = Neotropical, B = African, C = Indo-Malesian, D = Australian, E = Holarctic. The base map is sourced from the Global Administrative Areas Map (https://gadm.org/).

The newly-sequenced specimens were collected from 14 territories. Amongst them, 11 territories encompass the Planet’s major tropical forest zones: the Neotropics (Brazil, Colombia, Costa Rica, Guadeloupe, Peru, St Lucia) and the Palaeotropics (Madagascar, Mayotte, New Caledonia, Réunion, Taiwan) (Fig. 1). Almost all of these territories are located within major world biodiversity hotspots (Myers et al. 2000), including the so-called MIOI (Madagascar and the Indian Ocean Islands), the Caribbean (Guadeloupe, St Lucia), New Caledonia, the Colombian Chocó, Mesoamerica (Costa Rica) and the Atlantic coast of Brazil. The other newly-collected specimens came from Macaronesia (Madeira), East Asia (Taiwan) and Europe (Spain and the Netherlands). These specimens include both recent collections from 2021 to 2023 and some older specimens (i.e. collected in 2002) preserved in freezers. Detailed information on collection sites, collectors and vouchers are provided in the supplementary material (Suppl. material 1). After sampling, the specimens were carefully dried for several weeks using absorbent paper for foliicolous specimens and a box containing silica gel beads for non-foliicolous specimens. They were then stored at −20 °C until DNA extraction.

### ﻿DNA extraction, amplification and sequencing

Well-preserved and freshly collected specimens of *Gomphillaceae* (less than 6 months old or stored in the freezer) that showed no visible signs of fungal infection were selected for DNA extraction. The extractions were carried out in two laboratories in Belgium: the Botanical Institute at the University of Liège and the Meise Botanic Garden in Brussels.

The selection of markers and the PCR programme was guided by Xavier-Leite et al. (2022), who established the first phylogeny of the family based mostly on Brazilian foliicolous specimens and using two loci, the mitochondrial small subunit rRNA (mtSSU) and the nuclear large subunit rRNA (nuLSU). Nevertheless, modifications have been made to the previous DNA extraction protocol to overcome two major limitations: (1) low success rate after sequencing, 55% for nuLSU and 16% for mtSSU from a dataset of around 500 samples (pers. comm., R. Lücking); and (2) specimen loss, i.e. the frequent need to sacrifice entire specimens to obtain sequences (Xavier-Leite et al. 2022).

Given their usually small size and to avoid destroying entire specimens, the material was extracted using a Direct PCR approach. Small pieces of thalli (< 0.1 mm), setae or thin sections of apothecia were carefully removed and placed directly into 0.2 ml PCR tubes. The Sigma-Aldrich REDExtract-N-Amp Plant PCR Kit (St. Louis, Missouri, USA) was the most successful to obtain nuLSU (around 70% of success) and ineffective in obtaining mtSSU (less than 10% of success). This kit was used according to the manufacturer’s instructions, except that the extraction step was bypassed. The nuLSU was amplified using the primer pairs LR3 and LR0R (Vilgalys and Hester 1990) with the following PCR conditions: initial denaturation for 3 min at 95 °C, followed by 35 cycles of denaturation for 45 s at 95 °C, annealing for 45 s at 54 °C, elongation for 1 min at 72 °C and a final elongation for 10 min at 72 °C. The Direct PCR mix described in Ertz et al. (2015) was successful in obtaining mtSSU sequences (around 80% success rate). The primer pairs mrSSU1 and mrSSU3R (Zoller et al. 1999) were used with the following PCR conditions: initial denaturation for 10 min at 95 °C, followed by 35 cycles of denaturation for 45 s at 95 °C, annealing for 1 min at 52 °C, elongation for 75 s at 72 °C and a final elongation for 10 min at 72 °C.

Recognising that relying solely on two ribosomal markers limits the resolution and support of deeper phylogenetic relationships (Xavier-Leite et al. 2022), a protein-coding marker has been incorporated to enhance the reconstruction of the phylogeny’s backbone. We selected the largest subunit of RNA polymerase II (RPB1), due to its extensive use in phylogenetic studies of lichen families (Crespo et al. 2007; Magain and Sérusiaux 2014). Additionally, initial trials suggested that amplifying RPB1 was more straightforward than the second largest subunit of RNA polymerase II (RPB2), which has shown lower amplification success in related families like *Graphidaceae* (Rivas Plata et al. 2012). For instance, Miadlikowska et al. (2014) reported that the success rate for RPB1 was double that of RPB2. RPB1 amplification was performed using the Sigma-Aldrich REDExtract-N-Amp Plant PCR Kit, following the same protocol as for nuLSU, with, as a result, slightly better success rate than nuLSU (around 80%). The primer pairs RPB1Cr (Matheny et al. 2002) and RPB1-AFpelt (Hofstetter et al. 2007) were used with the following PCR conditions: initial denaturation for 3 min at 95 °C, followed by 35 cycles of denaturation for 45 s at 95 °C, annealing for 45 s at 50 °C, elongation for 1 min at 72 °C and a final elongation for 10 min at 72 °C. PCR products were visualised by electrophoresis on a 2% agarose gel, purified with VWR® ExoCleanUp FAST PCR (Radnor, PA, USA) and sequenced by Macrogen-Europe® (Maastricht, the Netherlands).

### ﻿Sequence editing and alignment

Forward and reverse sequence fragments were assembled using Geneious Prime v. 2022.2.2 (Biomatters, Auckland, New Zealand). Consensus sequences were then subjected to a BLASTn search (Altschul et al. 1997) in GenBank, using megaBLAST, to confirm their affiliation with the *Gomphillaceae*. Separate datasets for each locus were then assembled, incorporating sequences from GenBank. These sequences were selected primarily from Xavier-Leite et al. (2022), Miyazawa et al. (2022, 2023), Zhu et al. (2023) and Lücking et al. (2024) to determine the exact placement of the newly-sequenced taxa within a broader phylogeny of the family. Five accessions of *Fissurina* were selected as outgroup according to Xavier-Leite et al. (2022). In total, 2212 sequences (809 mtSSU, 956 LSU, 447 RPB1) corresponding to 1271 specimens were processed. Sequences were aligned using MAFFT v. 7 online (Katoh et al. 2019) and the alignments were checked manually with Mesquite v. 2023.3.81 (Maddison and Maddison 2023). Terminal ends of sequences and ambiguous regions of each dataset were delimited and excluded using the online version of Gblocks v. 0.91b (Castresana 2000) (http://phylogeny.lirmm.fr/), allowing for gap positions within the final blocks and carefully checked manually.

### ﻿Phylogenetic analysis

Analyses for topological incongruence amongst loci were performed on the *Gomphillaceae* dataset using a Maximum Likelihood (ML) approach with RAxML-HPC2 v.8.2.12 (Stamatakis 2014) on the CIPRES Web Portal (Miller et al. 2010). We evaluated models of DNA evolution for each locus with the programme jModelTest v.2.1.10 (Darriba et al. 2012) and the best models were chosen using the Akaike Information Criterion (AIC). For each locus, the GTRGAMMA model was employed and node support was assessed by running 1000 bootstrap replicates. Three ML trees were produced, one for each locus and the placement of specimens in each tree was compared. Topological incongruence was considered significant when conflicting relationships (monophyletic versus non-monophyletic) for the same set of specimens were both supported with bootstrap values ≥ 70% (Mason-Gamer and Kellogg 1996). Based on this criterion, we removed the following published sequences due to significant conflicts: GenBank: MZ827235 identified as *Actinoplacastrigulacea* was found to represent a species in the “Caleniaaff.graphidea 1” clade; GenBank: AY341363 identified as *Asterothyriumlongisporum* was actually a species of the genus *Monocalenia*; GenBank: MZ827230 identified as *Aulaxinaminuta* represented *Aulaxinaquadrangula*; GenBank: KF833327 identified as *Pseudocaleniasolorinoides* represented *Monocaleniamonospora*; GenBank: MZ827253 identified as “Gyalectidiumaff.imperfectum” clade represented *Gyalectidiumfilicinum*; GenBank: KF833351 identified as *Echinoplaca* sp. 6 represented a species in the genus *Gomphillus*; and GenBank: MZ827234 identified as Santrichariaaff.farinosa was actually *Rubrotrichasubhelminthospora*. After identifying and removing these conflicts, the mtSSU, nuLSU and RPB1 datasets were concatenated using the combine.pl script from the Plexus package (Magain 2018). The complete matrix contained 2207 sequences belonging to 1270 specimens (including outgroup) (Suppl. material 5). The completeness of the individual markers in the complete matrix was 63.5% for mtSSU, 75.5% for nuLSU and 35% for RPB1.

In cases where loci did not overlap and morphological characteristics were insufficiently discriminatory between specimens, a conservative approach was taken to prioritise morphology and the locality of origin. This conservative approach aimed to prevent the formation of artificial clades in the phylogenetic tree and avoid inflating species numbers. As a result, in nine cases, we concatenated sequences from distinct thalli into a single terminal in the matrix: *Aulaxinella* sp. nov. 3: EL2515a and EL2516b; *Caleniopsislaevigata*: LOT03-35207B and 23138; Trichariaaff.aulaxinoides (sterile 2): EL2548b and EL2543a; *Trichariaamazonum*: DE26526R and 22050; Adelphomycesaff.cochlearifer 6: DE27026B1 and DE27024B; Microxyphiomycesaff.demoulinii 2: EL1959b and 2382a; Echinoplacaaff.campanulata 1: EL1583a and EL1588b; *Vezdamycesalbopruinosus*: Aptroot56427 and 23046; *Vezdamycesalbopruinosus*: Aptroot56418 and 23052. In seven cases, they came from the same locality, including three cases from the same tree branch. Each of these nine cases has been discussed in detail in Suppl. material 5.

The matrix with these nine combined specimens contained 2207 sequences belonging to 1256 specimens (excluding outgroup) and served as the basis for species delimitation analyses. The alignment lengths were 1391 bp for mtSSU, 716 bp for nuLSU and 884 bp for RPB1 (Table 1). The best ML tree was reconstructed from the concatenated alignment using the same approach as previously. Phylogenetic trees were visualised using FigTree v. 1.4.4 (Rambaut 2018). Bootstrap values (BS) ≥ 70% were considered as relationships with high support.

**Table 1.** Summary of the complete matrix including number of specimens, number of analysed sites (before slash), total number before removing ambiguously aligned sites (after slash), number of variable characters, number of parsimony-informative characters and their respective proportions (in parentheses) for each locus separately. The outgroup is excluded.

Locus	Number of sequences	Number of char. Incl. / total number of sites	Number of variable char.	Number of parsimony-inf. char.
mtSSU	799 (0.64)	1072/1391 (0.77)	838 (0.78)	698 (0.65)
nuLSU	953 (0.76)	604/716 (0.84)	369 (0.61)	287 (0.48)
RPB1 1^st^ codon	445 (0.35)	210/210 (1.00)	115 (0.55)	97 (0.45)
RPB1 2^nd^ codon		209/209 (1.00)	90 (0.43)	71 (0.34)
RPB1 3^rd^ codon		209/209 (1.00)	205 (0.98)	204 (0.98)
RPB1 intron		255/255 (1.00)	187 (0.72)	165 (0.65)
RPB1 total		884/884 (1.00)	597 (0.68)	537 (0.61)

### ﻿Species delimitation methods

Four species delimitation methods were used to delimit Operational Taxonomic Units (OTUs), each representing a distinct species hypothesis. Three of them relied on molecular data for species delimitation: the Generalised Mixed Yule Coalescent approach (GMYC; Pons et al. (2006)), the Bayesian Poisson Tree Process (bPTP; Zhang et al. (2013)) and the Assemble Species by Automatic Partitioning (ASAP; Puillandre et al. (2021)). An integrative approach combining phenotypic data and molecular phylogenies was employed, taking into account the morpho-anatomical data of the specimens, their geographical origins and phylogenetic relationships. The candidate lineages were delineated using the phenotype matrix detailed in Xavier-Leite et al. (2024). This matrix, which allowed for the encoding of up to 233 phenotypic characters, served as a reference for comparing the lineages. The phenotypic data for each species were incorporated into this matrix and will be presented in Lebreton et al. (in prep.). The complete molecular matrix was divided into ten large clades to improve phylogenetic resolution and species delimitation results, each corresponding to separate datasets (Suppl. materials 2, 5). The molecular data-based analyses were performed: on the individual loci mtSSU and nuLSU for ASAP and GMYC and, additionally, on the 3-locus data (mtSSU-nuLSU-RPB1) for bPTP and GMYC.

For each dataset, ultrametric Bayesian trees (UB) and ML trees were generated. UB trees were generated for each subclade, on the mtSSU alignment alone, on the LSU alignment alone and on the three-locus alignment using BEAST v.2.6.6 (Bouckaert et al. 2014) on the CIPRES Science Gateway (Miller et al. 2010). For each alignment, an initial analysis was run for fifty million generations, sampling every 1000^th^ generation. In the three-locus analyses, clocks and trees were linked, whereas loci were unlinked. Lognormal relaxed clocks were used. Convergence of the runs was assessed using Tracer v. 1.7.1 (Rambaut et al. 2018). We considered that convergence was achieved when effective sample sizes (ESS) for all parameters was > 200. For runs that did not converge, we followed an iterative approach with increasing number of generations (100 million, 150 million, 300 million, 500 million) and testing both lognormal and exponential relaxed clocks, until convergence was reached. Burn-in was determined, based on ESS as visualised in Tracer. A maximum clade credibility tree was generated with TreeAnnotator v.1.8.2 from the posterior distribution of trees. The UB trees were used as input for GMYC for mtSSU, nuLSU and the combined 3-locus using the GMYC Web server (https://species.h-its.org/gmyc/) with default parameters and the single threshold method. As an input for bPTP, for each subclade, ML trees were generated with RAxML using the same parameters as described above. bPTP was run with the ML trees on the bPTP Web server (https://species.h-its.org/ptp) on the 3-locus data with the following settings: 500,000 MCMC generations, 100 thinning and 0.3 burn-in. The mtSSU and nuLSU matrix for ASAP was submitted separately to the ASAP web server (https://bioinfo.mnhn.fr/abi/public/asap/asapweb) using the K80 model. The transition to transversion ratios (TS/TV) were calculated in MEGA v. 11.0.10 (Tamura et al. 2021). The partition with the best ASAP score was selected for comparison with the other methods. The results from the four information sources were cross-checked for the 1256 specimens. In the case of conflicts between methods, a consensus was reached, based on the most frequently encountered delimitation. If no single delimitation was predominant, we followed the species delimitation hypothesis suggested by the integrative approach.

We generated an alignment consisting of one multilocus sequence per species (subset 1). For each species, we selected a specimen containing the three loci when possible, then two loci if possible, then one. Twelve cases of specimen combinations were considered, based on the results of species delimitation algorithms, which indicated, using at least one shared locus (with 99–100% of bp in common), that these specimens belonged to the same species. This approach aimed to increase the number of loci per species, thereby improving the resolution of the phylogenetic tree topology for ancestral area analysis (Suppl. materials 5, 7). As a result, two separate samples of the same species were combined: Actinoplacaaff.gemmifera: EL2707a and EL2731b; *Aderkomycespapilliferus*: DE27044b and DE27021a; Asterothyriumaff.microsporum 9: EL2365a and EL2366a, Caleniaaff.depressa 2: DE22043b and DE22000b; Caleniaaff.subdepressa 2: EL2546a and 2505a; Echinoplacaaff.pellicula 1: EL1594a and Aptroot86657; *Echinoplaca* aff. sp. nov. 13: DE27032A and DE27024F; *Echinoplaca* sp. nov. 15: EL2500b and EL2524a; *Echinoplaca* ‘sterile’ 6: EL2568a and EL2498a; *Gomphillushyalinus*: EL2294 and EL2290; *Gyalideopsis* sp. nov. 10: EL2554a EL2713a and Spinomycesaff.albostrigosus 3: EL1509 and EL838b. In these 12 cases, the specimens originated from the same territory, with eight cases specifically from the same locality.

After extracting the sequences for the 473 species from the complete matrix, the alignment was again revised and the ambiguous regions were delimited as previously explained. As a result, a total of 2627 bp (1113 bp for mtSSU, 628 bp for nuLSU and 884 bp for RPB1) were obtained for subset 1. The completeness of the individual markers was 77.5% for mtSSU, 76% for nuLSU and 59.5% for RPB1. This subset was used to estimate divergence times and to perform biogeographic analyses.

### ﻿Divergence time analysis

To test the rooting within *Gomphillaceae*, we filtered our dataset, removing species represented by a single locus from subset 1, resulting in a 349-species dataset (subset 2). We added an outgroup consisting of nine species with at least two loci in *Graphidaceae* retrieved from Lücking et al. (2013) (subset 3) for a total of 358 species (Suppl. material 1). The best ML tree was generated with RAxML-HPC2 using the same parameters as above. Subset 2 was also analysed with BEAST v.2.6.2 on the CÉCI cluster (https://www.ceci-hpc.be/about.html) using the same parameters as above. The analysis was run for 406.9 million generation sampling every 1000^th^ generation for a total of 406,943 trees. We removed 10% of trees as burn-in then selected one tree every 100^th^ tree for a total of 3996 trees. The maximum clade credibility tree was then generated on this subset using TreeAnnotator, with default parameters.

Then, we generated our final tree to perform divergence time analysis and biogeographical reconstruction by analysing subset 1 using BEAST v.2.6.6 on the CIPRES gateway. Loci were unlinked, but clocks and trees were linked. Lognormal relaxed clocks were used. The dataset was analysed with a topological constraint on the monophyly of all taxa, except *Gyalideasensu lato*, based on RaxML results (Suppl. materials 7, 8). The analyses were run with a calibration of 75 Mya (Mean 75, Sigma 0.1) on the root of the tree, following the estimates from Xavier-Leite et al. (2022). Trees were generated in three parallel runs with identical parameters, for a total of 689.86 million generations, sampling every 1000^th^ generation. Convergence was assessed using Tracer v.1.7.1, to determine the approximate number of generations at which log likelihood values stabilised and to identify the effective sample size (ESS) for each parameter. Trees from the three runs were combined using LogCombiner and 20% were discarded as burn-in. For computational reasons, we then selected a subset of our trees, sampling one tree every 15^th^ tree, for a total of 37,943 trees. The maximum clade credibility tree was then generated on this subset using TreeAnnotator, using default parameters.

### ﻿Biogeographical analysis

To measure the taxon overlap between geographical areas, we created a database documenting the presence or absence of species at three geographical levels: (1) foliicolous lichenogeographical regions according to Lücking (2003), (2) broad biogeographic regions corresponding to world floristic realms and (3) administrative subdivisions (countries or island) (Suppl. material 3). We first used the lichenogeographical regions of the world based on foliicolous lichen distributions as a base map to assign the records of *Gomphillaceae* to one of the six large regions defined by Lücking (2003) (Fig. 1). According to this classification, our study areas cover five large regions: New Caledonia and Taiwan are included in the Eastern Palaeotropics, Madagascar and the Mascarenes (Réunion, Mayotte) in the African Palaeotropics, Europe and Macaronesia (Madeira) in the Tethyan region and the Caribbean, South and Central America in the Neotropics (Fig. 1).

**Figure 2.**
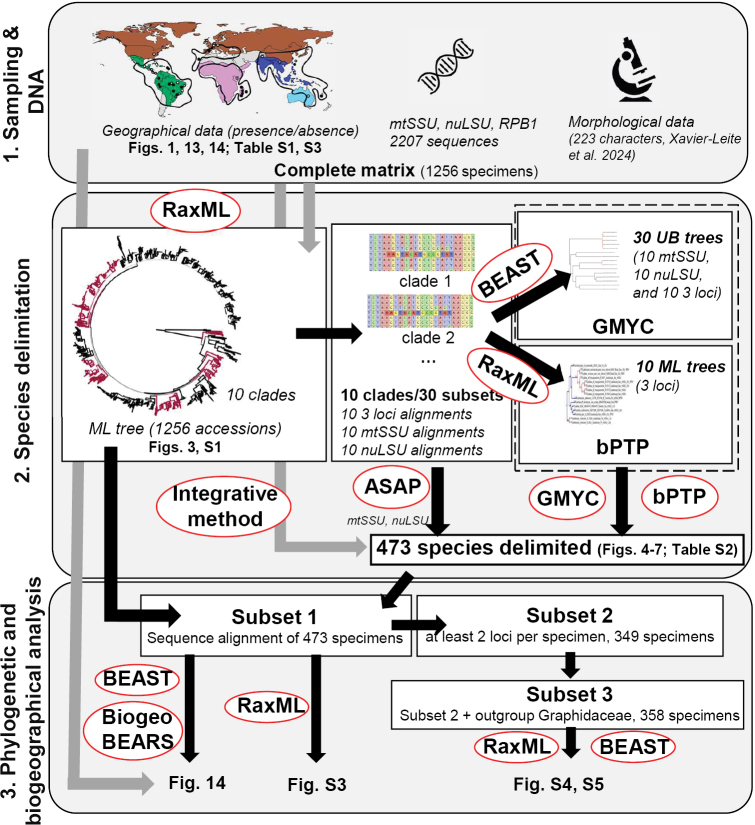
Graphical abstract of the Materials and Methods section.

Species were also coded into six broad biogeographic regions based on the floristic realms defined by Liu et al. (2023): Neotropical (A), African (B), Indo-Malesian (C), Australian (D) and Holarctic (E) (Fig. 1). At the administrative subdivisions scale, species were coded into 32 subdivisions corresponding to the studied territories. The number of species shared between two regions was calculated for the three geographical levels. The Sorensen similarity index (Sørensen 1948) was used to measure the overlap between territories based on the third delimitation (administrative subdivision areas). To reconstruct the geographic origins of *Gomphillaceae*, the floristic realms distribution was selected (Fig. 2).

We used the BiogeoBEARS package in R Studio (v. 4.4.0) (Matzke 2018) to reconstruct ancestral ranges and tested six models: Dispersal–Extinction–Cladogenesis (DEC) (Ree and Smith 2008), DEC+J (Matzke 2014), Dispersal–Vicariance Analysis (DIVAKAR), DIVAKAR+J (Ronquist 1997; Ronquist and Sanmartín 2011), BayArea-like (BAYAREALIKE) and BAYAREALIKE+J (Landis et al. 2013). The J parameter is associated with long-distance dispersal and founder-event speciation (Van Dam and Matzke 2016). We evaluated the relative probability of each model based on the Akaike Information Criterion (AIC). The analysis was conducted using our UB tree (subset 1) with the outgroup removed and took branch support into consideration for the interpretation of results. Given that the maximum number of biogeographic regions for a species in our sampling is five, the analysis was set to reconstruct a maximum of five ranges at ancestral nodes.

To determine if the over-representation of Neotropical taxa was influencing the results of BioGeoBears analyses, we generated five additional trees by removing 179 randomly-chosen species that are exclusively found in the Neotropics, retaining only 70 Neotropical species (we have 249 species exclusively found in the Neotropics, 70 in the Indo-Malesian, 70 in the Australasian and 67 in the African). The 179 species were randomly selected using the gshuf function in bash and this process was repeated five times. The selected taxa were then removed from the 473-species UB tree using the drop.tip function from the ape package (Paradis et al. 2004). BioGeoBears analyses were repeated on these five modified trees using the same parameters as before. All the methods used and the data subsets created are schematically summarised in Fig. 2.

### ﻿Predicting global species richness

The consensus estimate (E) for species numbers in the current dataset was used to predict the global richness of *Gomphillaceae*. First, we divided the consensus estimate into four categories: (a) known species, (b) hidden species (novel, cryptic or near-cryptic lineages emerging within presumably known taxa), (c) new species with novel phenotypes and (d) unidentified taxa (Suppl. material 2). We further assumed that our sampling covered 25% (ratio 4:1) of the total area of occurrence of *Gomphillaceae*. We then computed two ratios: (1) Species known prior to this study (459) vs. number of those species represented in the dataset (a), to estimate the global taxonomic coverage of the dataset; (2) the total of the hidden species (b) plus the corresponding known species divided by the latter, to estimate the ratio of hidden diversity. To estimate the global diversity of *Gomphillaceae* (G), based on these numbers and ratios, we proceeded as follows, assuming that additional unknown species must either represent new species with new phenotypes or further hidden diversity. Assuming that only 25% (ratio 4:1) of the global distribution area of *Gomphillaceae* was represented in the dataset (global vs. sampled; Suppl. material 6), we estimated the number of further new species to be discovered as 3 × (c), i.e. the total of new species to be expected [4 × (c)] minus those already in the dataset [1 × (c)]. In case of hidden diversity, we assumed that it would be greater in widespread species compared to those with narrow distribution. Therefore, we divided the remaining non-sequenced species [D = 459 – (a)] into three categories: pantropical (D_pantropical_), bicontinental and continental-wide (D_wide_) and continental-narrow (D_narrow_). Using the results from our dataset, we employed three mean ratios of hidden diversity: pantropical = R_pantropical_, bicontinental and continental-wide = R_wide_ and continental-narrow = 1 (no change). Since our dataset represented only 25% of the distribution range, our hidden diversity ratios also only applied to this limited range and so we further multiplied the first two ratios by 4. The total global diversity of *Gomphillaceae* was then estimated as G = E + D_narrow_ + 3 × (c) + 4 × [(D_pantropical_ × R_pantropical_) + (D_wide_ × R_wide_)] (Suppl. material 2).

In addition, we also estimated the extrapolated species richness for the three territories for which we had detailed locality information and the same sampling effort (Guadeloupe, New Caledonia and Taiwan), using species sample incidence frequencies based on the Chao2 estimator (Chao 1987) (27 localities for Guadeloupe, 20 for New Caledonia and 9 for Taiwan) with the R package iNEXT (Chao et al. 2014; Hsieh et al. 2016). We finally estimated the extrapolated species richness for *Gomphillaceae* at the global scale using incidence (presence/absence) data at the administrative subdivisions (countries or island) level (Suppl. material 3) on a global 32-territory dataset as well as on a reduced 14-territory dataset, using only territories where at least five species occur. We estimated the global species richness using the same approach as above with the iNEXT package and confirmed the result using the Chao 2 estimator (Chao 1987) with the specpool function of the vegan package in R (Oksanen et al. 2013).

## ﻿Results

### ﻿Specimens, sequence data and phylogenetic analysis

We generated a total of 1,707 new sequences from 913 specimens for this study: 628 mtSSU, 646 nuLSU and 433 RPB1 (Suppl. material 1). The completeness of the individual markers was 69% for mtSSU, 71% for nuLSU and 48.5% for RPB1. The lower coverage of RPB1 was not a result of amplification difficulties, but rather a decision to limit sequencing to selected specimens, unlike mtSSU and nuLSU. To improve the resolution of deeper phylogenetic branches with the addition of RPB1, we focused on two representative specimens per clade, as revealed by earlier phylogenetic analyses using mtSSU and nuLSU. Amongst the specimens, 231 had all three loci, 332 had two loci and 350 had one locus. Out of these, 213 specimens (414 sequences) were identified as belonging to formally described species, while 233 specimens (437 sequences) represented new species (identified on the basis of phenotype), 359 specimens (649 sequences) represented species complexes (hidden species with a phenotype corresponding to a described morphospecies) and 108 specimens (207 sequences) required taxonomic revision for identification or were poorly developed or sterile. The status of each specimen is detailed in the supplementary material (Suppl. material 2).

We analysed the three single-locus datasets for topological incongruence and since no conflict was detected, the nuLSU, mtSSU and RPB1 datasets were concatenated. Next, incongruences between the three-locus ML tree and the three-locus UB tree were examined. Both trees revealed three well-supported major clades (Suppl. materials 8, 9). Clade A represented the genus *Gyalidea*, clade B included the genera *Actinoplaca*, *Asterothyrium*, *Caleniella*, *Corticifraga*, *Linhartia*, *Psorotheciopsis* and *Taitaia* and clade C encompassed all other genera sequenced, such as *Adelphomyces* s.lat., *Aderkomyces* s.lat., *Aulaxina* s.lat., *Aulaxinella*, *Aptrootidea* s.lat., *Arthotheliopsis*, *Bastistomyces*, *Bezerroplaca* s.lat., *Bullatina* s.lat., *Calenia* s.lat., *Caleniopsis*, *Echinoplaca* s.lat., *Gomphillus*, *Gyalectidium* s.lat., *Gyalideopsis* s.lat., *Jamesiella* s.lat., *Lithogyalideopsis*, *Microxyphiomyces* s.lat., *Monocalenia*, *Paratricharia* s.lat., *Psathyromyces*, *Pseudocalenia*, *Roselviria*, *Rubrotricha*, *Rolueckia*, *Santricharia*, *Spinomyces*, *Sipmanidea*, *Sporocybomyces*, *Tricharia* s.lat., *Verruciplaca* and *Vezdamyces*.

The UB and ML trees differed in the placement of the *Gyalidea* clade (A), which represented the first split in the ML tree (Suppl. material 8) and a second split in the UB tree (Suppl. material 9). The UB tree showed a split between a clade formed by clades A and B and the clade C. The ML tree showed an initial split between clade A and the combined clades B and C. Since the relationship between A and B was not supported (pp = 0.58) in the UB tree, whereas the clade C was highly supported in the ML tree (BS = 85), we performed a new BEAST analysis with a constrained topology, forcing clades B and C to be monophyletic and, hence, *Gyalidea* as the first split. Within clade C, the foliicolous lineage *Rolueckia* forms the earliest split with strong support (pp = 1, BS = 100), followed by a clade consisting of the foliicolous genus *Rubrotricha* and the *Echinoplaca* sp. nov. clade (pp = 1, BS = 99).

### ﻿Species delimitation and global richness prediction

**Figure 3.**
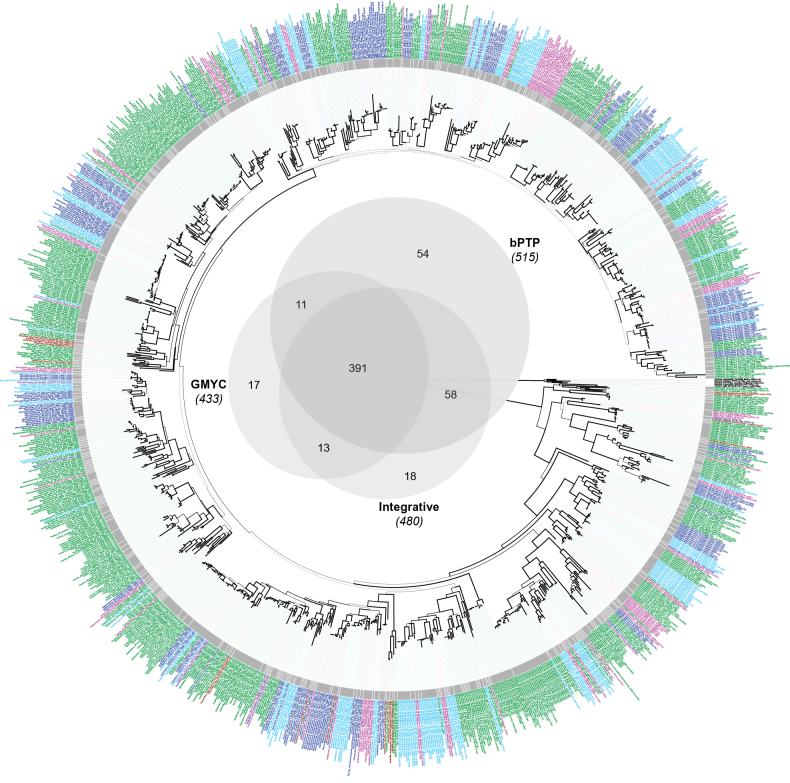
Global tree of *Gomphillaceae*, based on the mtSSU, nuLSU and RPB1 loci for a total of 1265 specimens. The tree is the best scoring Maximum Likelihood obtained from RaxML and the branches with bootstrap values ≥ 70 are thickened. In the centre, a Venn diagram illustrates the overlap between the bPTP, GMYC and Integrative approach species delimitation results on the 3-locus dataset. The consensus on species delimitation is represented by a grey circle outside the tree where each species is separated by white lines. Specimen labels are coloured using the same geographic colouring scheme as in Fig. 1. The figure can be enlarged to discern labels.

**Figure 4.**
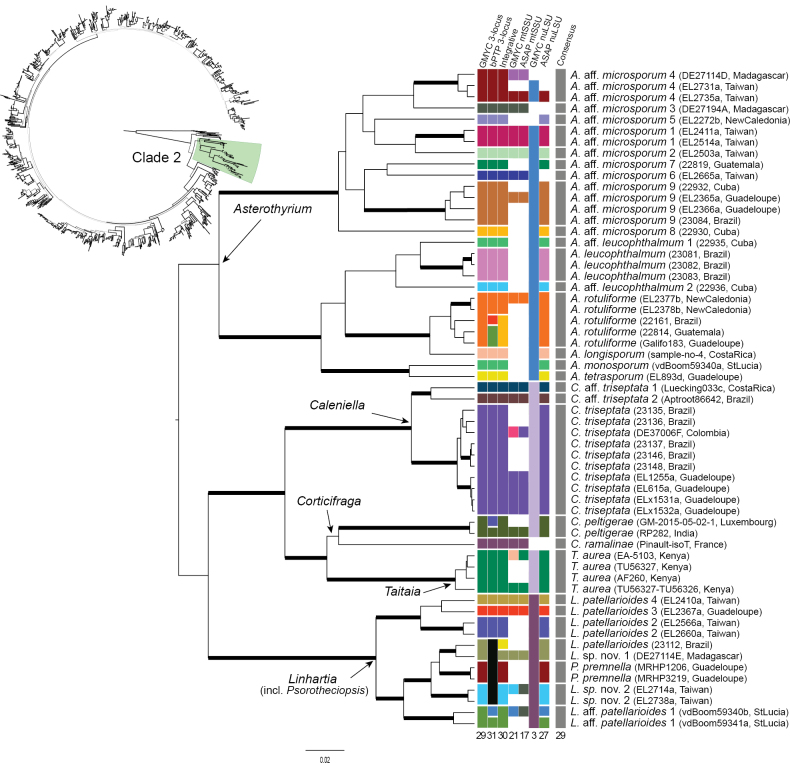
Results of the four species delimitation methods for clade 2 of the Ultrametric Bayesian (UB) tree obtained with BEAST. Top left: schematic representation of the global 3-locus tree of *Gomphillaceae* (see Fig. 4) highlighting clade 2. Centre and bottom right: chronogram resulted from the BEAST analysis on the 3-locus dataset on clade 2. Branches with posterior probability ≥ 0.95 are thickened. Centre: species delimitation results for each specimen presented in panels between the tree and the labels; from left to right: GMYC on the 3-locus dataset (mtSSU, LSU and RPB1, column 1), bPTP on the 3-locus dataset (mtSSU, nuLSU and RPB1, column 2), Integrative approach (column 3), GMYC on mtSSU (column 4), ASAP on mtSSU (column 5), GMYC on LSU (column 6), ASAP on LSU (column 7) and final consensus (column 8). Squares are represented in the same colour when specimens were reconstructed as part of the same species. Colours do not have other meaning besides shared species assignment. Total number of species reconstructed by each method is indicated below the column. Right: labels of the phylogenetic tree, representing the taxonomic assignment, voucher information and geographic origin, respectively.

**Figure 5.**
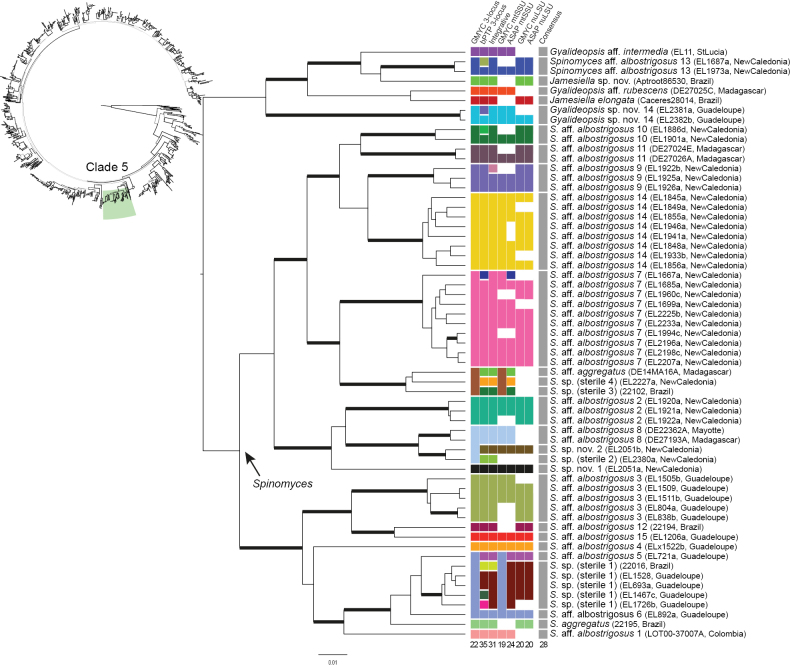
Results of the four species delimitation methods for clade 5 of the UB tree obtained by BEAST. Top left: schematic representation of the global 3-locus tree of *Gomphillaceae* (see Fig. 4) highlighting clade 5. Centre and bottom right: chronogram resulted from the BEAST analysis on the 3-locus dataset on clade 5. Branches with posterior probability ≥ 0.95 are thickened. Centre: species delimitation results for each specimen presented in panels between the tree and the labels; from left to right: GMYC on the 3-locus dataset (mtSSU, LSU and RPB1, column 1), bPTP on the 3-locus dataset (mtSSU, nuLSU and RPB1, column 2), Integrative approach (column 3), GMYC on mtSSU (column 4), ASAP on mtSSU (column 5), GMYC on LSU (column 6), ASAP on LSU (column 7) and final consensus (column 8). Squares are represented in the same colour when specimens were reconstructed as part of the same species. Colours do not have other meaning besides shared species assignment. Total number of species reconstructed by each method is indicated below the column. Right: labels of the phylogenetic tree, representing the taxonomic assignment, voucher information and geographic origin, respectively.

**Figure 6.**
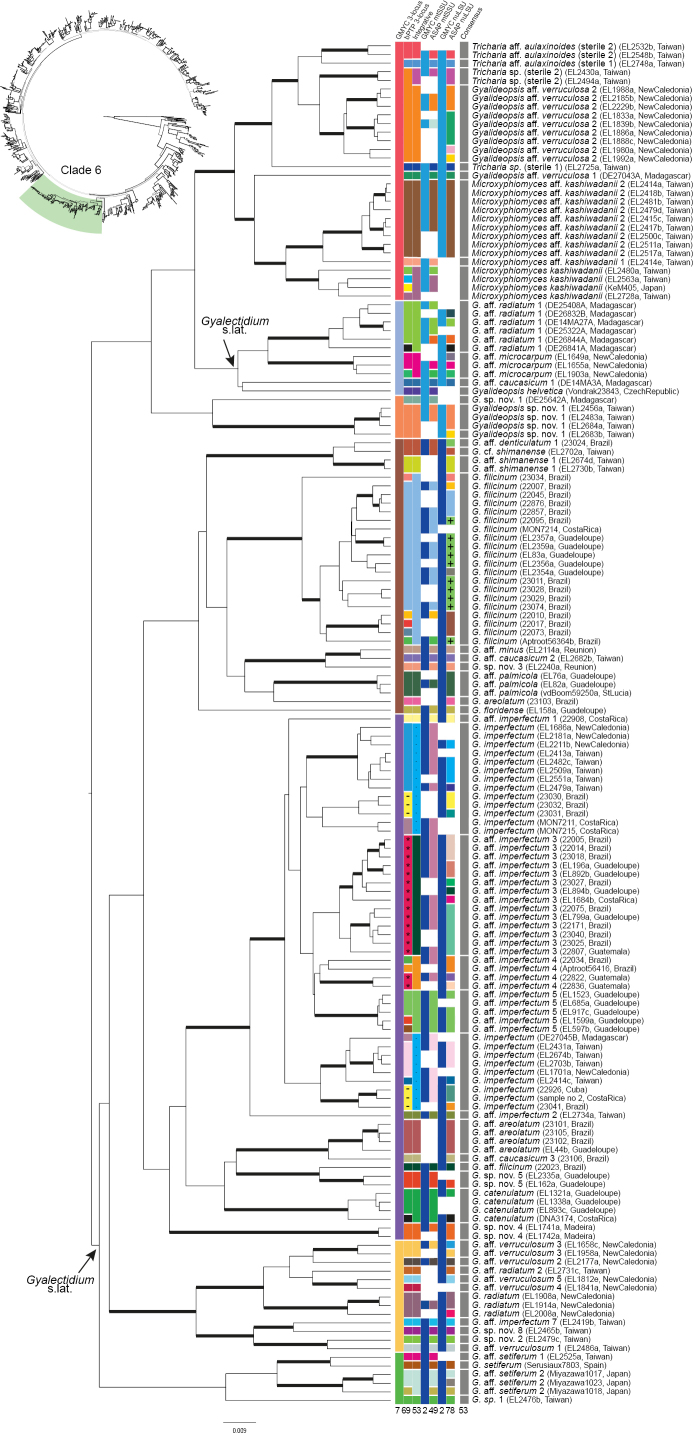
Results of the four species delimitation methods for clade 6 of the UB tree obtained by BEAST. Top left: schematic representation of the global 3-locus tree of *Gomphillaceae* (see Fig. 4) highlighting clade 6. Centre and bottom right: chronogram resulted from the BEAST analyses on the 3-locus dataset on clade 6. Branches with posterior probability ≥ 0.95 are thickened. Centre: species delimitation results for each specimen presented in panels between the tree and the labels; from left to right: GMYC on the 3-locus dataset (mtSSU, LSU and RPB1, column 1), bPTP on the 3-locus dataset (mtSSU, nuLSU and RPB1, column 2), Integrative approach (column 3), GMYC on mtSSU (column 4), ASAP on mtSSU (column 5), GMYC on LSU (column 6), ASAP on LSU (column 7) and final consensus (column 8). Squares are represented in the same colour when specimens were reconstructed as part of the same species. Colours do not have other meaning besides shared species assignment. Total number of species reconstructed by each method is indicated below the column. Right: labels of the phylogenetic tree, representing the taxonomic assignment, voucher information and geographic origin, respectively.

**Figure 7.**
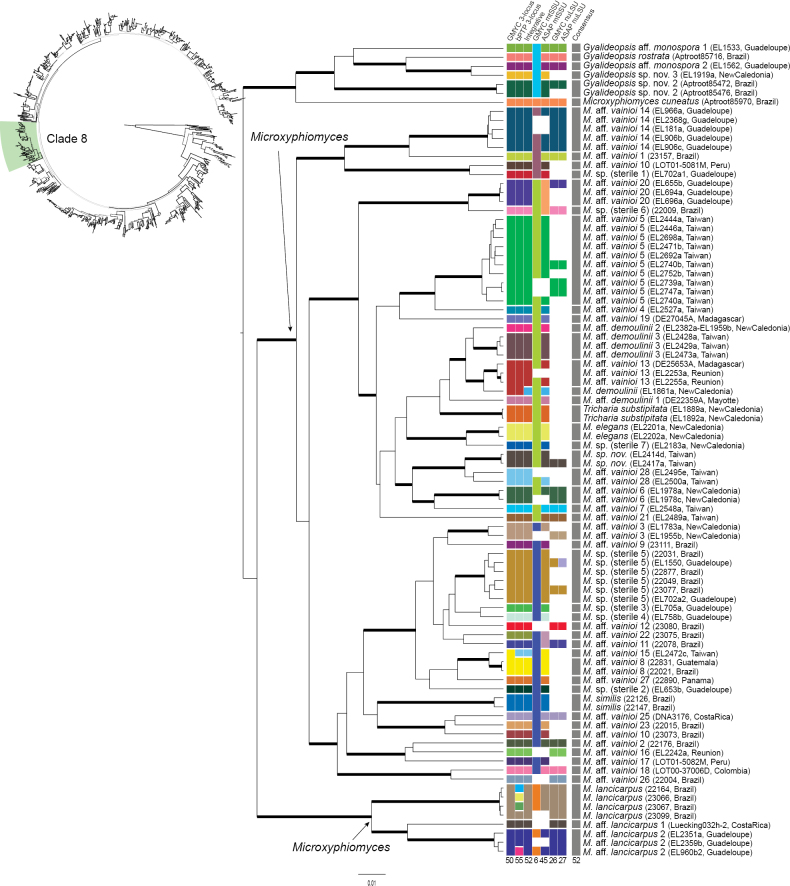
Results of the four species delimitation methods for clade 8 of the UB tree obtained by BEAST. Top left: schematic representation of the global 3-locus tree of *Gomphillaceae* (see Fig. 4) highlighting clade 8. Centre and bottom right: chronogram resulted from the BEAST analyses on the 3-locus dataset on clade 8. Branches with posterior probability ≥ 0.95 are thickened. Centre: species delimitation results for each specimen presented in panels between the tree and the labels; from left to right: GMYC on the 3-locus dataset (mtSSU, LSU and RPB1, column 1), bPTP on the 3-locus dataset (mtSSU, nuLSU and RPB1, column 2), Integrative approach (column 3), GMYC on mtSSU (column 4), ASAP on mtSSU (column 5), GMYC on LSU (column 6), ASAP on LSU (column 7) and final consensus (column 8). Squares are represented in the same colour when specimens were reconstructed as part of the same species. Colours do not have other meaning besides shared species assignment. Total number of species reconstructed by each method is indicated below the column. Right: labels of the phylogenetic tree, representing the taxonomic assignment, voucher information and geographic origin, respectively.

The number of hypothetical species, i.e. OTUs, delimited by each method, as well as the consensus on species delimitation for the 1,256 specimens, ranges between 433 and 515 (Fig. 3). The main cases of conflicts and agreements amongst the four methods are depicted for clades 2, 4, 6 and 8 (Figs 4, 5, 6, 7), respectively. The results of the remaining clade 1, 3, 5, 7, 9 and 10 are summarised in Suppl. material 2. The GMYC analysis based on the 3-locus dataset resulted in the delimitation of 433 OTUs (Fig. 3). The bPTP and integrative approach delimited 515 and 480 OTUs, respectively. In the single-locus analyses, ASAP delimited 391 OTUs for mtSSU and 489 OTUs for nuLSU. GMYC delimited 230 and 237 OTUs for mtSSU and nuLSU, respectively. ASAP lineages were split 9 and 11 times by GMYC in the mtSSU and nuLSU partitions. GMYC lineages were split 170 and 164 times by ASAP in the mtSSU and nuLSU partitions. Split of GMYC and integrative OTUs in the bPTP delineation generally reflected genetic variation amongst closely-related specimens from the same territory. For example, Spinomycesaff.albostrigosus 13 from New Caledonia split into two OTUs and *S.* “sterile 1” from Guadeloupe split into three OTUs in the bPTP analysis (Fig. 5). Divisions of GMYC by the integrative delineation or ASAP reflected the method’s lack of sensitivity to delimiting species in trees with short branches. For example, in clade 6, GMYC delimited 7 OTUs, while bPTP and the integrative approach delimited 69 and 53 OTUs, respectively (Fig. 6). GMYC in the single-locus analyses delimited less OTUs in the multi-locus partition. For instance, in the absence of RPB1 and mtSSU loci, the GMYC analysis failed to delimit species in the *Linhartia* and *Asterothyrium* genera (Fig. 4). GMYC conducted on the mtSSU locus in the clade 8 delimited 6 OTUs, while ASAP on the same dataset delimited 45 and GMYC on the multi-locus dataset delimited 50 (Fig. 7). Splitting of bPTP by other methods was particularly notable in clade 2. *Psorotheciopsispremnella*, *Linhartia* sp. nov. 2 and *Linhartiapatellarioides* were considered part of the same OTU according to the bPTP analysis, whereas GMYC and the integrative approach identified three distinct OTUs.

**Figure 8.**
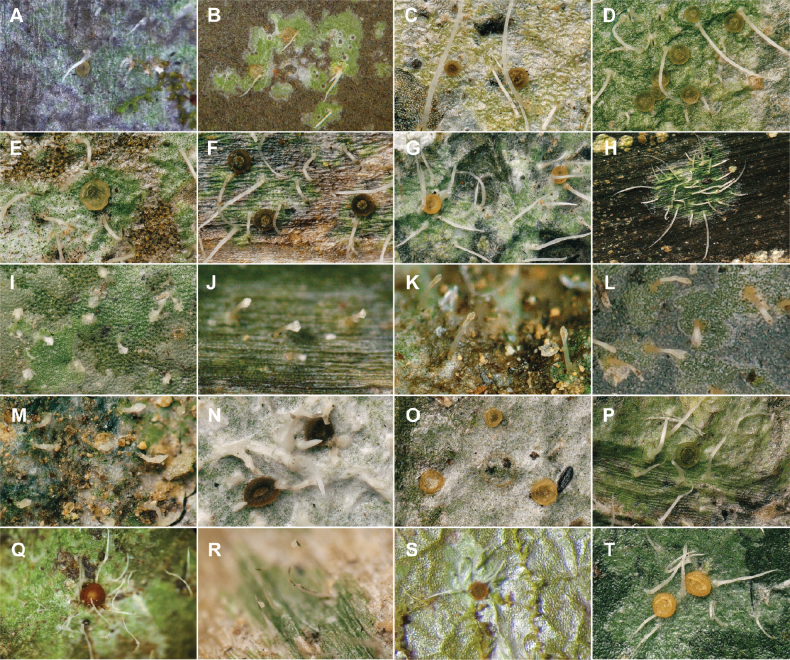
Selected specimens of the Spinomycesalbostrigosus morphotype and specimens representing clade 4 in topological order. **A**Spinomycesaff.albostrigosus 13 (EL1973a, New Caledonia), thallus with ascomata **B**S.aff.albostrigosus 10 (EL1901a, New Caledonia), thallus with ascomata and setae **C**S.aff.albostrigosus 11 (DE27026A, Madagascar), thallus with ascomata and setae **D**S.aff.albostrigosus 9 (EL1926a, New Caledonia), thallus with ascomata and setae **E**S.aff.albostrigosus 14 (EL1855a, New Caledonia), thallus with ascomata and setae **F**S.aff.albostrigosus 7 (EL2196a, New Caledonia) **G**S.aff.aggregatus (DE14MA16A, Madagascar) **H**S.aff.albostrigosus 2 (EL1922a, New Caledonia) **I**S.aff.albostrigosus 8 (DE22362A, Mayotte) **J**S.aff.albostrigosus 8 (DE27193A, Madagascar) **K***S.* sp. nov. 2 (EL2051b, New Caledonia) **L***Spinomyces* sp. (sterile 2) (EL2380a, New Caledonia) **M***S.* sp. nov. 1 (EL2051a, New Caledonia) **N**S.aff.albostrigosus 3 (EL804a, Guadeloupe) **O**S.aff.albostrigosus 15 (EL1206a, Guadeloupe) **P**S.aff.albostrigosus 4 (ELx1522b, Guadeloupe) **Q**S.aff.albostrigosus 5 (EL721a, Guadeloupe) **R**S.aff.albostrigosus 6 (EL892a, Guadeloupe) **S***S.aggregatus* (22195, Brazil) **T**S.aff.albostrigosus 1 (LOT00-37007A, Colombia). All are found on leaves, except **K** and **M**, on rock. Images by Lebreton and Ertz (**A–R, D**) and Xavier-Leite and Lücking (**S**).

**Figure 9.**
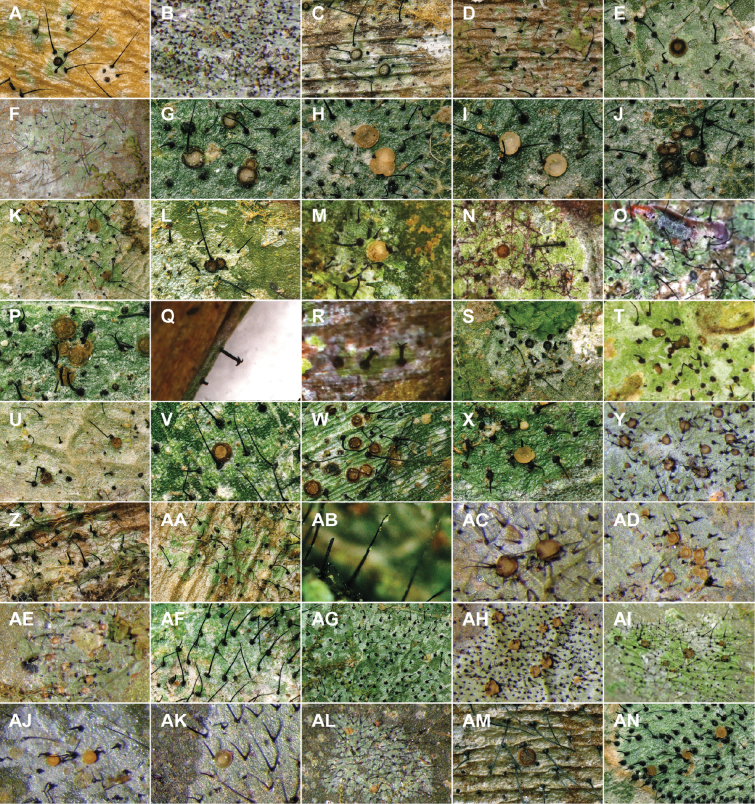
Selected specimens of the *Microxyphiomycesvainioi* morphotype and specimens representing clade 8 in topological order. **A**M.aff.vainioi 14 (EL2368g, Guadeloupe) **B**M.aff.vainioi 1 (23157, Brazil) **C**M.aff.vainioi 10 (LOT01-5081M, Peru) **D***M.* sp. (sterile 1) (EL702a1, Guadeloupe) **E**M.aff.vainioi 20 (EL655b, Guadeloupe) **F***M.* sp. (sterile 6) (22009, Brazil) **G**M.aff.vainioi 5 (EL2698a, Taiwan) **H**M.aff.vainioi 5 (EL2740b, Taiwan) **I**M.aff.vainioi 5 (EL2739a, Taiwan) **J**M.aff.vainioi 5 (EL2740a, Taiwan) **K**M.aff.vainioi 4 (EL2527a, Taiwan) **L**M.aff.vainioi 19 (DE27045A, Madagascar) **M**M.aff.demoulinii 3 (EL2473a, Taiwan) **N**M.aff.vainioi 13 (EL2255a, Reunion) **O***M.demoulinii* (EL1861a, New Caledonia) **P**M.aff.demoulinii 1 (DE22359a, Mayotte) **Q***Trichariasubstipitata* (EL1889a, New Caledonia) **R***M.elegans* (EL2201a, New Caledonia) **S***M.* sp. nov. (EL2414d, Taiwan) **T**M.aff.vainioi 28 (EL2500a, Taiwan) **U**M.aff.vainioi 6 (EL1978a, New Caledonia) **V**M.aff.vainioi 7 (EL2548a, Taiwan) **W**M.aff.vainioi 21 (EL2489a, Taiwan) **X**M.aff.vainioi 3 (EL1783a, New Caledonia) **Y**M.aff.vainioi 9 (23111, Brazil) **Z***M.* sp. (sterile 5) (EL702a2, Guadeloupe) **AA***M.* sp. (sterile 3) (EL705a, Guadeloupe) **AB***M.* sp. (sterile 4) (EL758b, Guadeloupe) **AC**M.aff.vainioi 12 (23080, Brazil) **AD**M.aff.vainioi 22 (23075, Brazil) **AE**M.aff.vainioi 11 (22078, Brazil) **AF**M.aff.vainioi 15 (EL2472c, Taiwan) **AG***M.* sp. (sterile 2) (EL653b, Guadeloupe) **AH**M.aff.similis (22147, Brazil) **AI**M.aff.vainioi 25 (DNA3176, Costa Rica) **AJ**M.aff.vainioi 10 (23073, Brazil) **AK**M.aff.vainioi 2 (22176, Brazil) **AL**M.aff.vainoi 26 (22004, Brazil) **AM**M.aff.vainioi 17 (LOT01-5082M, Peru) **AN**M.aff.vainioi 18 (LOT00-37006D, Colombia). Images by Lebreton and Ertz (**A, C, D, E, G–X, Z–AB, AF, AG, AM** and **AN**) and Xavier-Leite and Lücking (**B, F, Y, AC–AE, AH–AL**).

Two major conflicts between methods, due to tree topology conflicts in the ML and UB trees and unsupported relationships, were observed in clade 6 (Fig. 6), particularly in the *Gyalectidiumimperfectum* and the *G.filicinum* complexes. In these cases, the chosen consensus was conservative despite the identification of numerous OTUs by the ASAP method. In most cases (391 instances), there were no significant conflicts amongst the four methods and the delimitations reflected genetically close lineages, with the separation into distinct OTUs corresponding to changes in territory (Fig. 3). For example, the *Spinomycesalbostrigosus* complex was delimited into 19 OTUs, each belonging to a specific geographic region or floristic realm (Figs 5, 8). A similar biogeographic pattern was observed in the *Microxyphiomycesvainioi* complex (including the seven sterile *Microxyphiomyces*), which was delimited into 35 OTUs, all from distinct geographic regions (Figs 7, 9).

The consensus of the four methods on the 1,256 specimens estimates the total number of hypothetical species or OTUs in this dataset at (433–)473(–515). Our data encompass 104 (a) of the 459 formally described species of *Gomphillaceae*, leaving 355 species unsequenced. Amongst the 369 hypothetical species not formally named and identified by the four species delimitation methods, 213 (b) belonged to species complexes (hidden species, either fully cryptic or near cryptic) and 100 (c) were confirmed as new undescribed species. Finally, 56 (d) hypothetical species remain unidentified because the specimens are not sufficiently developed for certain identification. The 213 hidden species are associated with 69 morphospecies, including 18 with pantropical distributions, 10 with either bi-continental distribution and 17 with continental-wide distributions. To predict the total number of species, three mean ratios were computed and applied: one for pantropical morphospecies (R_pantropical_ = 7.5), another for bi-continental and continental-wide morphospecies (R_wide_ = 2.7) and a score of 1.0 for narrowly-distributed species. Amongst the 355 unsequenced species, 18 are pantropical, 44 are bi-continental, 28 are continental-wide and 265 are narrowly distributed. The mean of the total predicted global species richness was thus estimated at G = E + D_narrow_ + 3 × (c) + 4 × [(D_pantropical_ × R_pantropical_) + (D_wide_ × R_wide_)] = 473 + 265 + 3 × 100 + 4 × [(18 × 7,5) + (44 × 2,7) + (28 × 2,7)] = 2,356 species (Suppl. material 2).

**Figure 10.**
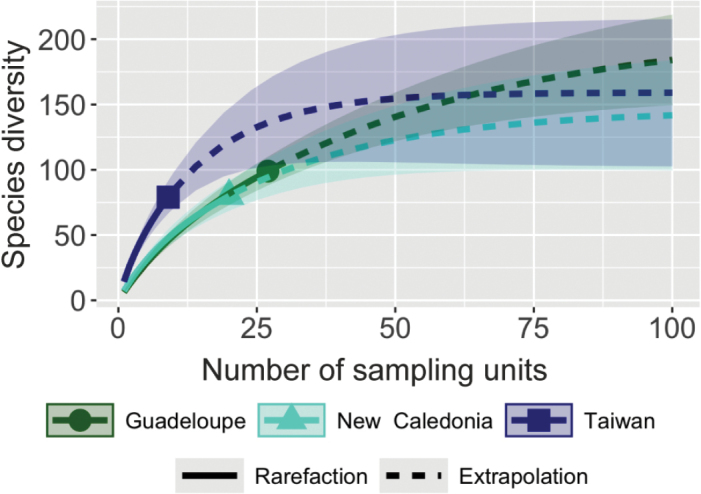
Rarefaction and extrapolation of species diversity curves as functions of the number of survey sites (sampling units), based on Hill’s numbers in Guadeloupe, New Caledonia and Taiwan.

**Figure 11.**
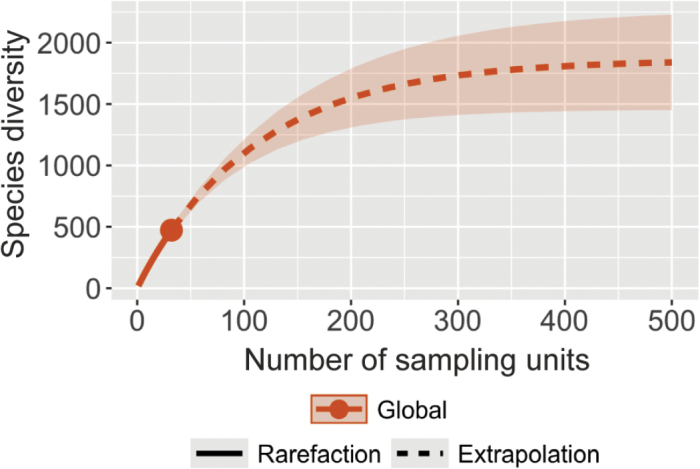
Rarefaction and extrapolation of global species diversity curve as functions of the number of territories (sampling units), based on Hill’s numbers.

Extrapolations, based on species sample incidence frequencies (Chao2 estimator), estimated a total of 207.8 (± 32.1) [estimated number (± standard error)] species for Guadeloupe (95% confidence interval 144.9–270.8), based on the distribution of 98 observed species in 27 localities; a total of 145.6 (± 22.4; 95 confidence interval 101.6–189.5) species for New Caledonia, based on the distribution of 80 species in 20 localities and a total of 159.1 (± 25.2; 95% confidence interval 109.7–208.6) species for Taiwan, based on 79 observed species in nine localities (Fig. 10). This estimate is only applicable to the northern part of Taiwan, since our sampling was only conducted in the north and different species are expected to occur in the southern part of the island. At the global level, the estimated number of species was 1861 (± 159.4) for the estimate based on 32 territories. The 95% confidence interval was 1549–2174 (Fig. 11). The estimate, based on the 14 territories with at least five species, was 1832.4 (±179.4). The 95% confidence interval was 1480.8–2183.9.

### ﻿Geographical patterns

Almost all the specimens studied (98%) were collected in the Tropics (Table 2). More than half of the species, 261 in total (55%) are at least Neotropical. The Palaeotropics, encompassing the African, Asian and Oceanian continents, represented 211 species, accounting for 45% of the total species diversity. The European, Mediterranean and North American regions were the least speciose, contributing only 1–3% of the recovered species. Brazil and Guadeloupe predominantly represented the Neotropics, accounting for 73% of the species in this floristic realm. These two territories comprised 40% of the total species diversity and 43.5% of the specimens in our dataset (Fig. 3). The Eastern Palaeotropics lichenogeographical region (Lücking 2003) was divided in Liu et al. (2023) into the Indo-Malesian floristic realm, primarily represented by Taiwan in our study and the Australian floristic realm, primarily represented by New Caledonia. These two realms had each 81 species (18% of specimens), collectively making up 45% of the species diversity. The African floristic realm was mainly represented by the MIOI (Madagascar and the Indian Ocean Islands), with 63 species, contributing 13% of the total species diversity (10% of specimens).

**Figure 12.**
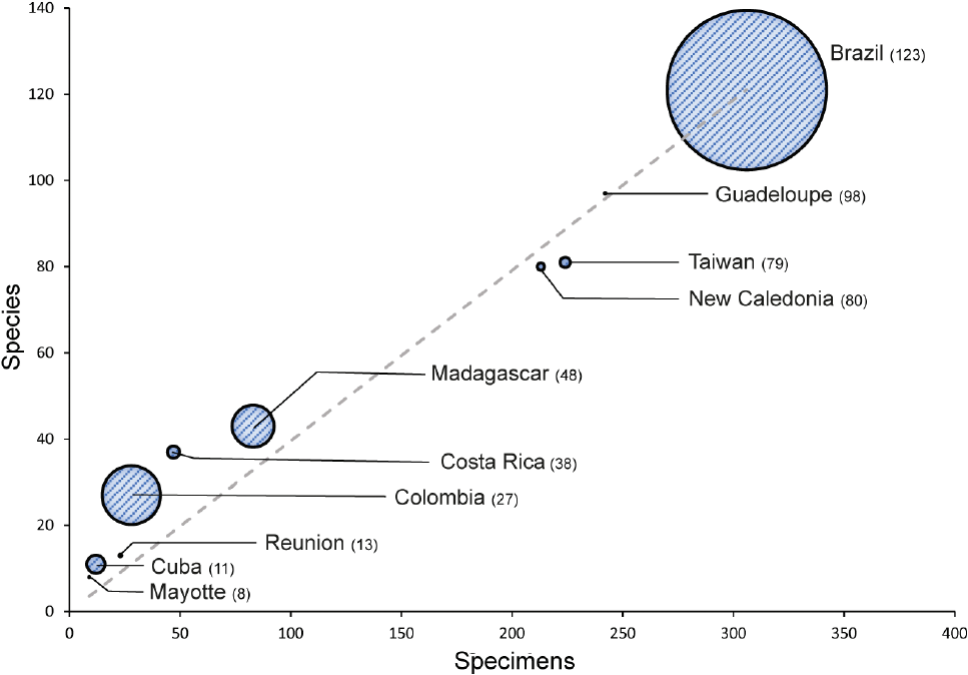
Comparison of species diversity relative to sequencing effort between the major sampled regions for *Gomphillaceae* collections. The dot size is proportional to the surface area of each territory. The dotted line represents the linear function y = x.

We analysed the number of specimens relative to the number of species and the area of the territories in the main collections studied (Fig. 12). There was an almost linear relationship between the number of specimens studied and the number of species found. The Pearson correlation test between these two variables indicates a correlation coefficient of 0.99 and a p-value of 2.2^e-16^ (Suppl. material 10). However, there is no correlation between the area of the territory and the species richness. For example, Guadeloupe, with an area of approximately 1,600 km² was one of the smallest territories studied, but had the second-highest number of *Gomphillaceae* species, with 98 species, just after Brazil, which had 123 species and an area 1,900 times larger than Guadeloupe. The fact that the number of species correlates much better with the number of specimens than with the area of the territory suggests that we are still very far from sampling the global diversity of these territories and that the total species richness must be much higher than what is included in our dataset.

**Table 2.** *Gomphillaceae* species diversity and number of specimens per area, lichenogeographical regions and floristic realms. *Note: The total number of species is 475, as two species are shared between tropical and temperate regions.

	Species # (%)	Specimens # (%)
*** Area ***		
Tropical	462* (97)	1236 (98)
Temperate	13* (3)	20 (2)
*** Lichenogeographical regions ***		
Neotropics	261 (55)	677 (54)
Eastern Palaeotropics	154 (33)	442 (35)
African Palaeotropics	63 (13)	119 (9.5)
Tethyan	8 (2)	10 (1)
outside	5 (1)	8 (0.5)
*** Floristic realms ***		
Neotropics	261 (55)	677 (54)
Indo-Malesian	81 (17)	228 (18)
Australian	81 (17)	214 (17)
African	63 (13)	119 (10)
Holarctic	13 (3)	18 (1)

**Figure 13.**
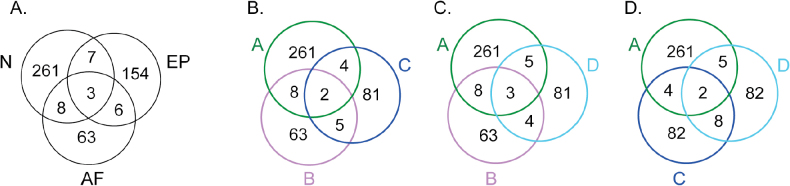
Venn diagrams illustrating the number of unique and shared species between: each geographical lichenogeographical regions defined by Lücking (2003) on the left and each floristic realm according to Liu et al. (2023) on the right. Diagrams coloured using the same geographic colouring scheme as in Fig. 1. N = Neotropics, EP = Eastern Palaeotropics, AF = African Palaeotropics. A = Neotropical, B = African, C = Indo-Malesian, D = Australian.

**Table 3.** Results of the BiogeoBEARS analysis. Each model implemented is presented with values for dispersal (d), extinction (e), founder (j), log-likelihood (LnL) and Akaike Information Criteria (AIC). The best-fitting model and its AIC value are highlighted in bold.

Model	d	e	j	LnL	AICc
DEC	0.005	0.004	0.000	-878.6161	1761.258
**DEC+J**	**0.001**	**0.000**	**0.05269627**	-**686.0147**	**1378.081**
DIVALIKE	0.006	0.001	0.000	-848.244	1700.514
DIVALIKE+J	0.001	0.000	0.05016653	-689.3034	1384.658
BAYAREALIKE	0.004	0.035	0.000	-1024.916	2053.858
BAYAREALIKE+J	0.001	0.000	0.05347292	-692.5356	1391.122

### ﻿Species and genus geographical overlap

The Sørensen similarity matrix revealed an overall low similarity (< 0.20) amongst the 32 administrative territories in terms of species overlap (Suppl. material 3), indicating that very few species are found in more than one territory. The highest level of similarity (1) was observed between India and Luxembourg, but this was due to insufficient sampling; both territories had only one known species, the lichenicolous *Corticifragapeltigerae*, which was present in both. A high similarity level (0.6) was also noted between Scotland and the USA, again due to a low number of known species in these territories. For regions with more than 10 species, a higher similarity was observed between territories within the same floristic realm. In the Neotropics realm, an index of 0.27, representing 31 shared species, was found between Guadeloupe and Brazil. Similarity indices also showed overlaps between Guadeloupe and Costa Rica (0.13), Guatemala and Brazil (0.13) and Costa Rica and Cuba (0.16). Within the African Palaeotropics, similarity was found between Madagascar and Mayotte (0.18). Despite having similar species diversity, Taiwan (79 species) and New Caledonia (80 species) shared only eight species. A significant portion of the species (397 species = 84%) was restricted to a single territory or country. Venn diagrams comparing foliicolous lichenogeographical regions defined by Lücking (2003) and floristic realms according to Liu et al. (2023), showed few common species between the major tropical areas colonised by *Gomphillaceae* (Figs 1, 3, 13). We did not find more species in common between the Indo-Malesian (C) and Australasian (D) realms, grouped in one lichenogeographical region named Eastern Palaeotropics by Lücking (2003), than between Africa (B) and the Neotropics (A) (Figs 1, 13). In fact, we found the same number of shared species, eight, between C and D and between B and A.

According to literature, 259 out of the 459 previously-known species of *Gomphillaceae* are foliicolous, with 29% presumed to have a widespread (intercontinental) distribution, including 10% classified as pantropical or cosmopolitan (Suppl. material 2). Amongst the 104 species with a valid name, confirmed and sequenced in our study, 32% are reported in literature as being distributed across 2–5 continents (lichenogeographical regions). Applying phenotype-based species concept on our specimens places this estimate at 40.5%. However, using our integrative approach to delineate species, which combines molecular and morphological data, this estimate is significantly reduced to 9%. Only three species were found across the three majors tropical lichenogeographical regions: *Echinoplacaepiphylla*, *Gyalectidiumimperfectum* and *Sporocybomycesleucotrichoides*. Only twenty species (4%) spanned two or three floristic realms. Nearly all *Gomphillaceae* species (457 species = 96%) are restricted to one lichenogeographical region and 453 species (95%) are restricted to one floristic realm (Table 2).

In general, we found little species overlap and substantial genus overlap (Figs 3–7). For example, the genera *Asterothyrium*, *Gyalectidium* s.lat., *Linhartia*, *Microxyphiomyces* and *Spinomyces* were found in multiple territories within the Palaeotropics and Neotropics. Amongst the 39 known and sequenced genera, only 14 were confined to a single floristic realm: *Actinoplaca*, *Bezerroplaca*, *Caleniella*, *Caleniopsis*, *Lithogyalideopsis*, *Paratricharia*, *Psathyromyces*, *Pseudocalenia*, *Rolueckia*, *Roselviria*, *Santricharia*, *Sipmanidea* and *Vezdamyces* for the Neotropics and *Taitaia* for the Palaeotropics.

### ﻿Ancestral area reconstruction

**Figure 14.**
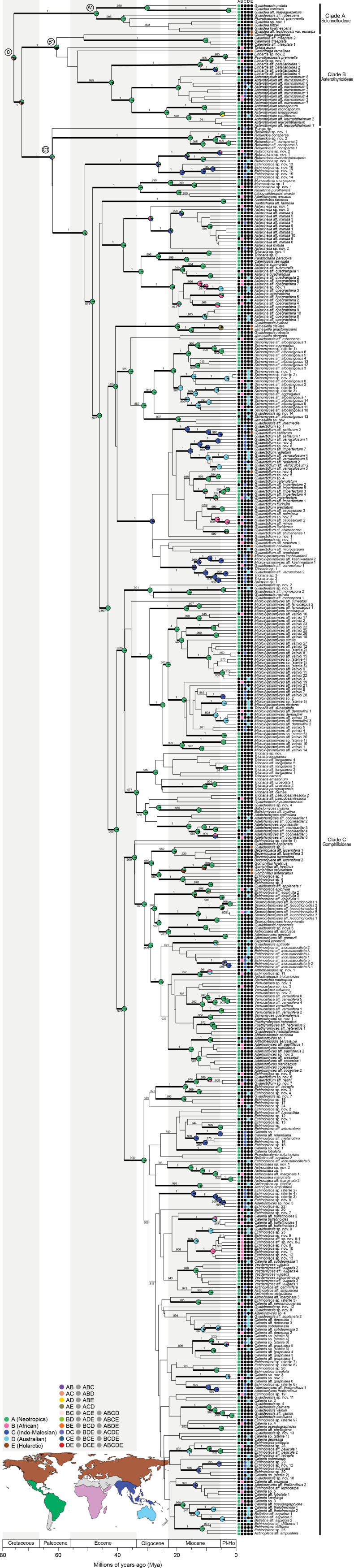
Result of the BioGeoBEARS analysis of *Gomphillaceae* in combination with the UB tree. Circles at nodes represent probabilities for ancestral areas resulting from DEC+J analysis and are plotted only for strongly-supported clades (posterior probabilities ≥ 0.95). Estimated ancestral area probabilities for selected nodes are given in Table 4 and in Suppl. material 4. Dots at the tips indicate extant *Gomphillaceae* species geographic area(s). World map (lower left) shows the five floristic realms.

The DEC+J biogeographical model was inferred with AIC as the best-fitting model for ancestral range estimation, based on the time-calibrated tree (Table 3; Fig. 14). According to this analysis, biogeographical patterns are mostly being driven by dispersal and founder-event speciation.

Our data indicated that the earliest intercontinental dispersal events occurred during the Oligocene (Fig. 14; nodes 5, 6 and 7) from the Neotropics to the Indo-Malesian realm. The majority of long-distance dispersal events took place during the Miocene, though they continued into more recent periods, including the Pleistocene and early Holocene. Successful long-distance dispersal events from the Neotropics to the Palaeotropics are estimated to have occurred at least 40 times, while 11 events in the reverse direction are inferred. The highest number of successful intercontinental dispersal events in our data were observed within the genera *Aulaxina*, *Asterothyrium*, *Gyalectidium*, *Linhartia* and *Rubrotricha*.

The pie chart for the *Gomphillaceae* crown node (node 0) indicated the highest probability for a Neotropical origin (42%), followed by a probability for an origin in either Asia or the Neotropics (24%) (Fig. 14). Node A1, corresponding to the subfamily *Solorinelloideae*, showed a Neotropical origin with a very high probability (99%). Node B1, representing the *Asterothyrioideae* crown node, suggested a Neotropical origin with a probability of 54%. Finally, the *Gomphilloideae* crown node (node C1) also pointed to a Neotropical origin with a probability of 51%. Most well-supported genera in our phylogeny had a predominantly Neotropical origin, with exceptions such as *Aulaxinella*, *Gomphillus* and *Rubrotricha*, which was Palaeotropical, with Indo-Malesian origins for *Aulaxinella* and *Rubrotricha* and a Holarctic origin for *Gomphillus*. However, our reconstructions may be biased by the over-representation of Neotropical taxa in our dataset. The impact of the over-representation of Neotropical taxa, tested five times on our entire dataset, resulted in five alternative reconstructions regarding the ancestral area probabilities of the lineages (Tables 3, 4; Suppl. material 4). The BiogeoBEARS analyses for node 0, corresponding to the *Gomphillaceae* crown node, produced two scenarios with a clear Neotropical origin and three scenarios where the origin is uncertain, involving the Neotropics, Indo-Malesia and Holarctic Regions (Table 4).

In scenarios described by subsets 2 and 3, the second highest probability included both the Neotropics and Indo-Malesian. In the scenario described by subset 5, the origin was shared between Indo-Malesian and Holarctic. Results for the five subsets also varied for the ancestral areas of the subfamilies *Asterothyrioideae* and *Solorinelloideae*, corresponding to nodes A1 and B1, respectively (Suppl. material 4). The *Solorinelloideae* crown node indicated a Neotropical origin in four scenarios with very high (95% and 84%) or moderate probabilities (40% and 41%). The analysis on subset 5 indicated a 100% Indo-Malesian origin. For the *Gomphilloideae* crown node (node C1), the BiogeoBEARS analyses across the five subsets showed four scenarios suggesting a Neotropical origin with an average probability of 50%, consistent with the results for the entire dataset (51%) (Table 5).

The last scenario indicated an Indo-Malesian origin with a probability of 40%, closely followed by a 39% probability for the Neotropics.

**Table 4.** Estimated ancestral area probabilities for node 0, corresponding to the *Gomphillaceae* crown node, as resulted from the BiogeoBEARS analysis. The dataset includes the complete database (Fig. 14) and each subset corresponding to the random removal of 179 species exclusively found in the Neotropics. The floristic realms are illustrated in Fig. 14 and coded as follows: A, Neotropics; B, African; C, Indo-Malesian; D, Australasia; E, Holarctic. The combinations of these realms are AB, AC, AE, BE, CE, ABC, ABE, ACE, ADC, BCE, ABCE, ABDC, ADCE and ABDCE. The first highest ancestral area probability value is highlighted in bold and italics, followed by the second highest in bold.

Realm	Dataset	Subset 1	Subset 2	Subset 3	Subset 4	Subset 5
A	***42***%	***33***%	10%	12%	***34***%	2%
C	1%	3%	3%	3%	2%	4%
E	0%	1%	7%	7%	1%	16%
AB	3%	5%	1%	2%	5%	0%
AC	**24**%	**25**%	13%	12%	**19**%	2%
AE	6%	7%	**15**%	**18**%	13%	11%
BE	0%	0%	1%	1%	0%	2%
CE	0%	1%	11%	10%	1%	***25***%
ABC	4%	8%	3%	3%	5%	1%
ABE	2%	2%	2%	3%	2%	3%
ACE	11%	10%	***21***%	***21***%	10%	**18**%
ADC	0%	0%	0%	0%	1%	0%
BCE	0%	0%	2%	2%	0%	5%
ABCE	6%	4%	5%	5%	3%	8%
ABDC	0%	0%	0%	0%	1%	0%
ADCE	0%	0%	1%	0%	0%	1%
ABDCE	0%	0%	1%	0%	0%	1%

**Table 5.** Estimated ancestral area probabilities for node C1, corresponding to the *Gomphilloideae* crown node, as resulted from the BiogeoBEARS analysis. The dataset includes the complete database (Fig. 14) and each subset corresponding to the random removal of 179 species exclusively found in the Neotropics. The floristic realms are illustrated in Fig. 14 and coded as follows: A, Neotropics; B, African; C, Indo-Malesian; D, Australasia; E, Holarctic. The combinations of these realms are AB, AC, BC and ABC. The first highest ancestral area probability value is highlighted in bold and italics, followed by the second highest in bold.

Realm	Dataset	Subset 1	Subset 2	Subset 3	Subset 4	Subset 5
A	***51***%	***50***%	***44***%	***49***%	***56***%	**39**%
B	0%	3%	2%	2%	2%	3%
C	**25**%	**28**%	**35**%	**32**%	**24**%	***40***%
AB	0%	1%	1%	1%	1%	1%
AC	23%	13%	15%	14%	13%	12%
BC	0%	1%	1%	0%	1%	1%
ABC	0%	3%	2%	2%	2%	2%

## ﻿Discussion

### ﻿Ever-expanding phylogeny of the Gomphillaceae

This study provides the first comprehensive worldwide molecular phylogeny of *Gomphillaceae*, a major component of foliicolous lichen communities (Lücking 2008). Our study significantly expands on previous data (Xavier-Leite et al. 2022), increasing the molecular sampling of this family by +235% at the species level (from 141 to 473). The phylogeny has nearly quadrupled in size, with a +264% increase in terminal branches (from 347 to 1265).

We improved the phylogenetic resolution, especially at the backbone of the phylogeny which previously lacked support (Xavier-Leite et al. 2022). This allowed us to clarify the phylogenetic placement of the former families *Asterothyriaceae* and *Solorinellaceae*, which were previously considered para- or polyphyletic due to insufficient support and limited taxon sampling. Moreover, the placement of some terminals was found to be based on concatenation errors. For instance, the inclusion of mtSSU sequences from other representatives of the genera *Asterothyrium* and *Monocalenia* revealed that the placement of *Asterothyrium* in Xavier-Leite et al. (2022) was based on a single mtSSU sequence (GenBank: AY341363), which belonged to *Monocalenia* (Lücking et al. 2004). Similarly, the putative placement of *Psorotheciopsis* (GenBank: MZ851727) with *Gyalidea* was based on a single sequence from a poorly-developed specimen putatively identified as *P.premnella* (Xavier-Leite et al. 2022). New sequences from fertile specimens have now clarified the placement of this taxon within the *Linhartia* clade (former *Asterothyriaceae*), where it was expected to belong (Henssen and Lücking 2002). The former families *Solorinellaceae*, *Asterothyriaceae* and *Gomphillaceae* are now resolved as monophyletic with strong support, although all three are phylogenetically closely related, with a long, shared stem branch. Within the broader *Gomphillaceae*, the first split is the clade formed by the genus *Gyalidea*, corresponding to the former family *Solorinellaceae*. The second split delimits a clade consisting of the genera *Asterothyrium*, *Caleniella*, *Cladosterigma*, *Corticifraga*, *Linhartia*, *Psorotheciopsis* and *Taitaia*, corresponding to the former family *Asterothyriaceae* plus some additional, newly-recognised genera, while the remaining genera belong to *Gomphillaceae* in the strict sense. The previously suggested differences, such as the absence of hyphophores and the unbranched paraphyses in *Solorinellaceae* and *Asterothyriaceae* vs. the presence of hyphophores and anastomosing paraphyses in *Gomphillaceae* s.str., are thus confirmed.

Our findings confirm that the *Gyalidea* clade corresponds to the earliest split within the *Gomphillaceae* s.lat. as proposed by Lücking et al. (2005) and Dennetière and Péroni (1998). Additionally, they confirm that the foliicolous lineage *Rolueckia* is the first split in the *Gomphillaceae* s.str., consistent with the findings of Xavier-Leite et al. (2022). Genera such as *Aderkomyces*, *Calenia*, *Echinoplaca*, *Gyalideopsis* and *Tricharia*, previously identified as polyphyletic (Lücking et al. 2005; Xavier-Leite et al. 2022), are confirmed as such in our study, in part with additional lineages not previously recognised. Genera previously recovered as monophyletic, such as *Gyalectidium* and *Jamesiella*, were resolved as paraphyletic in our expanded dataset, although the corresponding topology did not receive support and requires further study. Additionally, the placement of the genus *Lithogyalideopsis* is now established, but there are still six genera with no sequence data available: *Aplanocalenia* Lücking, Sérus. & Vezda, *Diploschistella* Vain., *Ferraroa* Lücking, Sérus. & Vezda, *Hippocrepidea* Sérus., *Paragyalideopsis* Etayo and *Phyllogyalidea* Lücking. The current phylogeny thus includes 39 described genera and dozens of new lineages outside the currently-described genera. The significant increase in the number of recognised genera within this family since Santesson (1952) is, therefore, expected to continue, potentially reaching the preliminary estimate of 60 genera discussed by Xavier-Leite et al. (2022). The formal re-instatement of the three former families — *Solorinellaceae*, *Astero­thyriaceae* and *Gomphillaceae* — as subfamilies along with the new genera identified in our study will be published in an upcoming paper.

### ﻿Undiscovered and hidden diversity and global richness of *Gomphillaceae*

Our DNA sequence data reveal an increase in the species diversity of the *Gomphillaceae* family, from 104 to 473 species. The extension of sampling to little-known regions, particularly in the Palaeotropics, has led to the discovery of over 100 species new to science and 213 belonging to species complexes, indicating outstanding hidden diversity in this family. The discovery of additional species is expected to continue, with vast tropical and subtropical areas in continental Africa, Asia and Australia yet to be explored. In addition, our data show a linear relationship between the number of specimens studied and the number of species discovered, indicating that many species remain undiscovered even within the territories already studied. Three field expeditions dedicated to collecting *Gomphillaceae* were conducted in Guadeloupe, New Caledonia and Taiwan, resulting in Chao’s estimations of 208, 146 and 159 species respectively. However, actual species diversity could be higher than these estimates, especially in New Caledonia and Taiwan, both of which are large islands with only a small fraction of forests having been explored.

The extensive sequence data now available for this family have uncovered hidden diversity within species previously thought to be well understood, with cryptic species to morphospecies ratios close to or exceeding 10:1 such as the lichenicolous *Adelphomycescochlearifer* (7:1), *Asterothyriummicrosporum* (9:1), *Aulaxinaopegraphina* (13:1), *Aulaxinellaminuta* (11:1), *Microxyphiomycesvainioi* (28:1) and *Spinomycesalbostrigosus* (19:1). Most of these morphospecies reflect genetically close lineages, with the differentiation into presumed species corresponding to changes in territory, continent or realm, but also often subtle details in morphology. For instance, the widespread collective taxon known as Microxyphiomyces (Tricharia) vainioi shows distinct, yet taxonomically unexplored variation in the length and density of the sterile thallus setae, the shape, size and colour patterns of the apothecia and the shape and size of the ascospores. Another case is *Asterothyriummicrosporum*, which rarely occurs fertile and so specimens with pycnidia only are usually subsumed under this name, but may well belong to taxa that differ in apothecial and ascospore features.

In other families, some foliicolous taxa considered to be widely distributed demonstrate a much more restricted range after a careful phenotype re-examination. Even without molecular data, the populations previously considered to represent *Trichotheliumminus* Vain. in Australia (Santesson and Tibell 1998) were later identified as *T.javanicum* (F. Schill.) Vězda (Lücking et al. 2001). Similarly, the Neotropical population of *Tapellariabilimbioides* R.Sant. (Santesson 1952) was identified as a new species, *T.albomarginata* Lücking (Lumbsch et al. 2011). The foliicolous lichen *Strigulasmaragdula* Fr. is a particularly notable example. Originally described from Nepal, Santesson (1952) had included Neotropical, Palaeotropical and temperate European populations within a broad concept of the species. Already without molecular data, the name *Strigulabuxi* Chodat was re-instated for the European populations by Roux and Sérusiaux (2004). Recent molecular phylogenetic studies focusing on (sub)tropical Asia demonstrate that *S.smaragdula* s.lat. is actually a species complex with substantial cryptic diversity (Jiang et al. 2017a, 2017b, 2022; Woo et al. 2020). This complex displays a well-structured phylogeny that correlates with phenotypic characteristics and regional and local distribution ranges (Ford et al. 2019; Jiang et al. 2020, 2022).

Species-level phylogenetic diversity in the family *Gomphillaceae* was here assessed using various species delimitation approaches, whose applications have been extensively discussed (Lumbsch and Leavitt 2011; Yeates et al. 2011; Fujita et al. 2012; Carstens et al. 2013; Leavitt et al. 2016b; Thines et al. 2018; Hausdorf and Hennig 2020; Huang 2020; Jorna et al. 2021; Padial and De la Riva 2021). Comparable to other works, (e.g. Dal-Forno et al. (2022)) in our study, bPTP exhibited a tendency to oversplit, identifying 515 species in the dataset. In contrast, GMYC did not separate singleton sequences into species, particularly in the *Gyalectidiumimperfectum* and *Microxyphiomycesvainioi* complexes, likely due to numerous short branches indicating recently diverged species. Algorithms, such as GMYC and bPTP, which identify differences in the branching rates of phylogenetic trees, are affected by gene flow (Jackson et al. 2017; Sukumaran and Knowles 2017; Luo et al. 2018). For GMYC, rapid, recent radiations may lead to inaccurate results (Reid and Carstens 2012; Wei et al. 2016; Myllys et al. 2023), while bPTP can overestimate species with strong intraspecific genetic structure (Leaché et al. 2019). Species delimitation methods are also sensitive to two key parameters: (1) geographic sampling (Mason et al. 2020) and (2) the number of loci analysed (Dupuis et al. 2012). Missing samples in contact zones between localities and low number of loci could lead to an overestimation of species richness (Chambers et al. 2023; Dufresnes et al. 2023). In our study, we analysed specimens from a limited number of territories worldwide using only three loci (mtSSU, nuLSU and RPB1). These two primary limitations are closely tied to the nature of foliicolous lichens. The DNA of foliicolous representatives degrades quickly, making it challenging to extract DNA from specimens collected more than six months earlier. Given current extraction protocols, our study was constrained to freshly-collected specimens, significantly limiting the geographic scope of our sampling. Furthermore, the minuscule size of these lichens (averaging 1 mm in diameter and less than 0.1 mm in thickness) and our direct PCR approach restricted the number of loci we could obtain. Moreover, to avoid completely destroying the specimens (thallus), we relied on Sanger sequencing (Direct PCR), which limited our capacity for more extensive genomic analysis, but allowed us to obtain sequences from 913 specimens. Future studies should test our species hypotheses by incorporating data from regions located between the areas we sampled. Generating data for numerous loci from thalli across hundreds of specimens is a promising challenge for future research on these tiny lichens.

In light of these limitations in species delimitation based on our molecular data, the integrative approach provides a balanced method by combining phenotypic, ecological and genetic data (Grube and Kroken 2000; Maharachchikumbura et al. 2021). This approach looks at monophyletic clades and their structure under the aforementioned species delimitation methods and then maps phenotypic characters, ecology and distribution on to the subclades. For instance, a clade split into several units by bPTP, but with no perceptible differences in any other feature between those units would likely be considered a single species in an integrative approach. In contrast, units further supported by phenotypic or ecological differences would be hypothesised to represent different species. Units differing only in distribution range could be considered species or subspecies, depending on branch length patterns and distribution ranges. However, while this approach intends to reach more stable, broadly supported solutions, it faces limitations when the relationships between lineages lack support and the phenotypic traits are not distinctive between specimens. This issue is evident in the *Gyalectidiumimperfectum*, *G.filicinum* and *Sporocybomycesleucotrichoides* complexes, where species delimitation algorithms, based on mtSSU, LSU and RPB1, may lack robustness. In our study, a conservative approach was used for species delimitation in these two complexes, in the spirit of Carstens et al. (2013). Incorporating additional loci into our dataset or employing alternative methods (e.g. involving NGS data) is anticipated to enhance species delimitation in such cases (Dupuis et al. 2012; Boluda et al. 2019). The ITS marker has been widely used in species delimitation models for various fungal groups due to its greater variability compared to LSU, mtSSU and RPB1 (Schoch et al. 2012). In highly speciose genera, the ITS region has played a crucial role in identifying specimens belonging to species complexes, as recently demonstrated with *Ramalina* (Jorna et al. 2021; Blázquez et al. 2024). Unfortunately, few ITS sequences are available for *Gomphillaceae*, whose initial phylogeny was based on mtSSU and nuLSU loci (Xavier-Leite et al. 2022). Amplifying ITS proved challenging for many foliicolous specimens within this family. The success rate was consistently low and seemed to vary depending on the clade targeted. Concentrating efforts on specific genera may offer a more effective approach for obtaining ITS sequences.

Although diagnostic characters may be subtle, our data indicate that many of the previously unrecognised species-level lineages are not fully cryptic. Rather, morphological characters now crucial for differentiating species were overlooked in historical studies on *Gomphillaceae*. For instance, Santesson’s monograph on foliicolous lichens (1952) did not recognise hyphophores as belonging to these lichens and, so, this information was not used in diagnoses. He also adopted a relatively broad concept in thallus or apothecial features, often because only few specimens were available to assess variation. With the recognition of hyphophores as asexual reproductive organs of *Gomphillaceae* by Vězda (1973), these structures have become diagnostic for distinguishing species and genera within this family (Sérusiaux and De Sloover 1986; Vězda and Poelt 1987; Sérusiaux 1998; Ferraro et al. 2001; Ferraro 2004; Lücking et al. 2005, 2006, 2007; Xavier-Leite et al. 2018, 2022, 2023; Miyazawa and Ohmura 2024). The possibility of deriving large character matrices including thallus, apothecial and hyphophore features has allowed the assessment of the putative placement of non-sequenced *Gomphillaceae* using phenotype-based phylogenetic binning (Xavier-Leite et al. 2024). The matrix of 223 phenotypic characters used in this previous study represents a valuable complementary approach to strengthen the species boundaries highlighted by our study and describe new species.

The global richness prediction of *Gomphillaceae*, based on estimated sampling effort and estimated species richness in the dataset, including hidden diversity and new species with novel phenotypes, resulted in an estimate of 2,356 species. The range of uncertainty in this estimate is largely given by the variation in the species delimitation methods, ranging from 433 (lowest) to 473 (mean) to 515 (highest) and the uncertainty of the estimation of the geographic range represented by the data, here assumed to be 25% (ratio 4:1 when extrapolated globally), perhaps with a variation of between 33% (ratio 3:1) and 20% (ratio 5:1). This would result in a combined uncertainty level of roughly 25%, translating into a prediction range of (1,767–)2,356(–2,945) species of *Gomphillaceae*. The assumed range of sampling effort of (33–)25(–20)%, resulting in a ratio of (3–)4(–5):1 in area increase when a global sampling effort would be undertaken, is supported by a comparison of the present study with Xavier-Leite et al. (2022). The latter was largely based on sampling from limited areas in Central and South America, with most samples from Brazil (260) and Costa Rica (36), i.e. the Neotropics, whereas the current study added larger numbers of samples for the Caribbean (Guadeloupe: 243), Madagascar (84), Taiwan (226) and New Caledonia (214), i.e. adding the African and the Eastern Palaeotropics, besides additional collections from other regions. The area increase of roughly 3:1 (three vs. one tropical regions) aligns well with the increase of mean species estimates in the two datasets, from 141 to 473 (a ratio of 3.35:1). The apparent linearity in the increase of richness with area is explained by the finding of our study that most species are regional endemics (see below), so additional broad sampling in still understudied areas, such as continental Africa and Asia, Indonesia and Oceania, but also the northern and central Andes in South America, justifies an assumed further increase by a ratio of (3–)4(–5):1. The alternative estimate, based on the Chao2 estimator and species sample incidence frequencies from 32 territories, suggests the existence of 1,861 species within this family, with a 95% confidence interval of 1,549–2,174 (± 159). This is roughly 20% lower than the estimate of (1,767–)2,356(–2,945) species. The difference suggests that the global area extrapolation ratio for the first estimate should perhaps be closer to 3:1 instead of 4:1.

These new predictions of roughly 2,000 species for the *Gomphillaceae* nearly double the previous estimate by Xavier-Leite et al. (2022), who suggested that the family could comprise 850–1,300 species in roughly 60 genera, with an average species-to-genus ratio of 20:1. Notably, our current analysis suggests up to 120 genera, also doubling the estimate by Xavier-Leite et al. (2022), so this is in line with roughly doubling their species level estimate. *Gomphillaceae* could, thus, be one of the most diverse families of lichen-forming fungi, lining up with *Graphidaceae* and second only after *Parmeliaceae* (Lücking et al. 2017a). However, it must be pointed out that especially the *Graphidaceae*, but also other species-rich families, such as *Arthoniaceae*, *Hygrophoraceae*, *Lecanoraceae*, *Peltigeraceae*, *Ramalinaceae*, *Teloschistaceae* and *Verrucariaceae*, either lack such broad species-level assessments or have many unknown species recognised in molecular data, yet not validly described. Therefore, some of these families are expected to perhaps reach levels of species richness comparable to *Parmeliaceae*, *Graphidaceae* and *Gomphillaceae*.

### ﻿Strong geographical patterns of diversity

About 98% of our data concerns foliicolous lichens and the Neotropics exhibit the highest known diversity in the *Gomphillaceae*. However, our results suggest that the Palaeotropics harbour greater foliicolous lichen diversity than previously thought, for example, in Lücking (2003). We collected approximately 3000 leaves during the field trips in Guadeloupe, Taiwan and New Caledonia. With similar sampling efforts, the species count was almost identical between Taiwan (79 species, 224 specimens) and New Caledonia (80 species, 214 specimens) and slightly higher in Guadeloupe (18–19 additional species, 242 specimens). Furthermore, we have not yet reached completeness regarding the species diversity of *Gomphillaceae* in any of these territories, suggesting that we have only seen the ‘tip of the iceberg’. Additionally, several important areas, such as continental Africa and continental Asia, as well as Indonesia and the Philippines, have not been included in our analysis.

The distribution of species is mostly restricted to one territory and usually does not expand beyond floristic realms as defined by Liu et al. (2023) or foliicolous lichenogeographical regions defined by Lücking (2003). Exceptions to this pattern include *Echinoplacaepiphylla*, *Gyalectidiumimperfectum* and *Sporocybomycesleucotrichoides*, which appear to be genuinely pantropical. This result challenges previous understandings of foliicolous lichen biogeography in our study territories. Traditionally, it was commonly accepted that foliicolous lichens have broad distributions across floristic realms and exhibit low levels of endemism (Sérusiaux 1989; Lucking and Kalb 2001; Lücking 2003; Lücking et al. 2003a, 2009; Herrera-Campos et al. 2004). For instance, approximately 68% of species in Guadeloupe were considered pantropical (Santesson 1952; Vězda and Vivant 1992; Sérusiaux 1998; Bricaud 2009), while in New Caledonia, this figure raised 64% (Lücking and Kalb 2001). In Réunion, around 80% of foliicolous lichens were thought to be pantropical (Sérusiaux 1977; Rønhede et al. 2003; Van Den Boom et al. 2011). Within the Neotropics, Cáceres et al. (2000) demonstrated that approximately 85% of Brazilian foliicolous lichens (Atlantic Rainforest) overlap with territories in Central America and 66% are widely distributed species occurring at least on two continents. The comprehensive morphological analysis of approximately 800 foliicolous species conducted by Lücking (2003) revealed that 57–70% of them are found in at least two of the three primary tropical regions: Neotropics, African Palaeotropics and Eastern Palaeotropics.

In contrast to all previous studies, our data on the *Gomphillaceae* family indicates that only 1–3% of species are shared across these regions, indicating high endemism at a continental scale. Lichen fungal taxa traditionally thought to have wide distributions, such as *Asterothyriummicrosporum*, *Aulaxinaopegraphina*, *Microxyphiomycesvainioi* and *Spinomycesalbostrigosus*, have been found to be species complexes comprising species with restricted distributions. These findings are consistent with studies that have uncovered significant cryptic speciation associated with restricted geographical distributions, as observed in genera such as *Acantholichen* (Dal-Forno et al. 2016), *Cora* (Lücking et al. 2014), *Dendriscosticta* (Simon et al. 2022), *Flavoparmelia* (Del-Prado et al. 2013), *Graphis* (Kraichak et al. 2015), *Lecanora* (Zhang et al. 2022), *Peltigera* (Magain et al. 2023) and the family *Parmeliaceae* (Divakar et al. 2016; Leavitt et al. 2016b).

As circumscribed here, 84% of the species have distributions restricted to one territory out of the 32 territories studied. However, the limited species overlap observed between territories may be due to insufficient sampling and should be further tested by adding new data from the gap regions. Ten of the studied territories represent islands (Cuba, Guadeloupe, Iriomote, Madagascar, Madeira, Mayotte, New Caledonia, Réunion, St. Lucia and Taiwan), accounting for 70% of the specimens studied and 67% of the species diversity (315 species). It would be interesting, in the context of obtaining more data from continents, to test the hypothesis of more restricted endemism within the different lichenogeographical regions. Indeed, island systems are known to host high endemism rates amongst lichens such as in the genera *Nephroma* (Sérusiaux et al. 2011), the *Ramalinadecipiens* complex (Blázquez et al. 2024) and the *Xanthoparmeliasubramigera* complex (Pérez-Vargas et al. 2024) in Macaronesia, the genera *Pseudocyphellaria* and *Sticta* in Hawaii (Moncada et al. 2014, 2020), the genera *Acantholichen*, *Cora*, *Cyphellostereum* and *Dictyonema* in the Galapagos (Dal-Forno et al. 2017). Several studies on macro-lichens have been conducted in our study territories and discuss significant endemism rates, as seen in molecular-based research on the genus *Sticta* in the Caribbean (Mercado-Díaz et al. 2023) and in the MIOI (Simon et al. 2018) which highlighted many species restricted to a single island. A recent comprehensive study involving 625 specimens (36 species) of the genus *Parmotrema* demonstrated that 70% are endemic to the MIOI, with 50% strictly endemic to Réunion (Masson et al. 2024). Our specimens come from some of the world’s most critical biodiversity hotspots, like Madagascar or New Caledonia whose flora exhibit 80–90% of taxa characterised by short-range endemism (Pellens and Grandcolas 2009; Véron et al. 2021; Antonelli et al. 2022). Our findings suggest that floristic realms, which offer a more restrictive zonation than traditional lichenogeographical regions, particularly by distinguishing the Indo-Malesian from the Australian Regions, might better represent the biogeographical patterns of foliicolous lichens.

### ﻿Long distance dispersion and rapid diversifications

The *Gomphillaceae* and the subfamily *Gomphilloideae* likely originated in the Neotropics, as nearly all analyses indicate the Neotropics as the source, either alone or in combination with one or two other realms, even when a substantial number of Neotropical taxa are excluded from the datasets. However, this result may be biased, as the earliest diverging lineage, the former *Solorinellaceae*, with the genus *Gyalidea* s.lat., has not been well sampled and is largely extratropical and non-foliicolous. A Neotropical origin of the family could be related to major extinction events affecting ancestral foliicolous lineages from the Holarctic Region (the Holarctic diversity loss hypothesis) (Meseguer and Condamine 2020). The evolutionary history of the *Gomphillaceae* must be linked to (sub-)tropical rainforests (megathermal forests), which provide the preferred habitat for existing foliicolous representatives. Studies indicate that all extant foliicolous lineages originated after the K–Pg boundary 66 million years ago (Mya) and diversified mainly during the Miocene (Xavier-Leite et al. 2022). This period was marked by overall cooling and a rapid temperature decline, causing megathermal rainforests to retract to lower latitudes (Morley 2007). These forests disappeared from most of North America (northern “boreotropical” region) and became much more restricted in Europe. Given that foliicolous lichens are sensitive to environmental changes (Lücking 1997b), it is likely that these abrupt changes caused extinctions and subsequently limited the dispersal of foliicolous *Gomphillaceae* towards northern latitudes. Extratropical lineages like *Gyalectidiumsetiferum* from Europe and *Gyalectidium* sp. nov. 4 from Madeira have a young phylogenetic age and are derived from primarily Neotropical ancestral lineages. Some early lineages may have survived by colonising other substrates, such as rocks and bark. The first dispersal events are estimated to have occurred around 54–57 Mya and involve the majority of non-foliicolous taxa, including the lichenicolous *Corticifraga* and *Taitaia* lineages. This could support the hypothesis of the extinction of Holarctic ancestral foliicolous lineages following the disappearance of megathermal forests. The geographical origins of the different clades and the paths of dispersal should be interpreted cautiously due to the lack of data from major regions such as the African continent, Australia and Malaysia. Future studies should focus on acquiring data from these areas to better understand the biogeographical history of the *Gomphillaceae*.

Regardless of where the family originated, our results strongly support numerous events of relatively-recent long-distance dispersal followed by subsequent speciation events. This results in the presence of numerous species with restricted ranges within genera with much broader distributions. A pattern of vicariance would have resulted in much more geographical structure for older events, deeper in the tree and more homogeneous geographic origins within more recent clades. Our data indicate that most dispersal events occurred during the Miocene, coinciding with the increased diversification rates observed in this family during that time. Given that little plate tectonics have taken place since this period, the Miocene disjunctions identified in our analysis are more effectively explained by long-distance dispersal rather than vicariance. If disjunct *Gomphillaceae* populations were remnants of continuous pre-Miocene distributions, we would expect to see biogeographically structured clades with longer divergence times, rather than intercontinental lineages sharing similar haplotypes across disjunct populations. Furthermore, the insular occurrences largely represented by our data, especially concerning oceanic islands, demonstrate the ability of *Gomphillaceae* to disperse over long distances, either through their vegetative propagules, such as conidia from pycnidia and diahyphae from hyphophores (Ferraro 2004) or by ascospores (Lücking 2008). New Caledonia is the most isolated territory in our study, leading us to hypothesise that its foliicolous lichen composition results from long-distance dispersal rather than vicariance or short-distance dispersal. This hypothesis is supported by the fact that the nearest territories, the Vanuatu Archipelago, is located about 540 km away and emerged in the Miocene, while New Caledonia was fully submerged during the post-rift Maastrichtian–Early Paleocene period (75–60 Mya) (Maurizot and Campbell 2020). However, since the theory of New Caledonia’s complete submersion remains a topic of ongoing debate (He et al. 2016; Giribet and Baker 2019; Malem et al. 2023), vicariance and short-distance dispersal cannot be entirely ruled out.

Successful long-distance dispersal event is evidenced by the numerous clades shared with either the Neotropics or the Palaeotropics. These are likely the result of dispersal to suitable habitats both in the distant and more recent past. Intercontinental gene flow has already been demonstrated for spore-dispersed organisms such as non-lichenised fungi (Moncalvo and Buchanan 2008), mosses (Lewis et al. 2014) and ferns (Weigelt et al. 2015). This phenomenon is supported by phylogeographic studies on lichen species such as *Leptogiumfurfuraceum* (Otálora et al. 2010), *Lichenomphaliaumbellifera* (Geml et al. 2012), *Psoradecipiens* (Leavitt et al. 2018b), *Porpidiaflavicunda* (Buschbom 2007) and *Xanthoparmeliapulla* (Amo De Paz et al. 2012). Long-distance dispersal events and diversification during the Miocene have also been considered crucial in shaping the global distribution of *Leptolejeunea*, an epiphyll liverwort genus sharing a similar ecological niche as foliicolous lineages of *Gomphillaceae* (Bechteler et al. 2017; Shu et al. 2022).

The argument for vicariance versus long-distance dispersal in foliicolous lichens is traditionally based on the fact that rainwater is the principal dispersal vector, with diaspores usually dispersed over short distances (< 1 m) (Lücking 2001; Lücking and Bernecker-Lücking 2002). Although the dispersal capacities of these lichens have not yet been tested through population genetics, several factors support the existence of long-distance dispersal events in foliicolous lichens. Experimental cultures developed by Sanders and Brisky (2022) demonstrated that *Gomphillaceae* of the *Gyalectidium* genus can disperse by discharging their diaspores into the air, independent of water’s mechanical action and subsequently germinating. Given that wind is considered a major vector for diaspore dispersal in many fungi (Golan and Pringle 2017), these diaspores could be transported over long distances by jet streams, particularly during storms and hurricanes. Additionally, migratory birds (Lewis et al. 2014; Garrido-Benavent et al. 2018) or complex dispersal scenarios involving step-wise migration along tropical highland bridges (Fernandez-Mendoza and Printzen 2013) may facilitate the transport of propagules or plant fragments bearing lichens. Like diaspores, asexual propagules in *Gomphillaceae* are likely quite resilient. Conidia produced by hyphophores (called diahyphae) from specimens collected in Guadeloupe were successfully cultured in our laboratory in Belgium over 5 weeks after collection, despite spending all this time under dry condition and in the cold (0–10 °C) during transport and storage. Additionally, foliicolous lichens are not very specific about the tree species they colonise (Lücking 1998), allowing them to easily establish in different tropical forests as long as the conditions of temperature, sunlight and humidity are suitable for their development. Interestingly, our results indicate that the highest number of successful intercontinental dispersal events occurs in *Gyalectidium* lineages. Many representatives of this genus co-disperse both partners during asexual reproduction: the algae are released alongside their diahyphae through the hyphophores. Recently, the ability of these lichens to discharge ascospores with attached epihymenial algae was demonstrated by Sanders and Brisky (2022) considering the algae associated with *Gomphillaceae* could then provide valuable insights on biogeographic patterns in this family. Therefore, future studies on foliicolous lichens should focus on more in-depth population genetics, including photobiont, with denser sampling targeting specific clades or species complexes.

Long-distance dispersal events are likely rare and followed by rapid allopatric speciation, given the limited number of species shared between continents, the significant genetic variation observed within genera and individual morphospecies and species exhibiting initial splits dating back to the late Pleistocene and Miocene. Several hypotheses can be proposed to explain this phenomenon. The short generation times linked to the ephemeral nature of the substrates colonised by these lichens (Lücking 2008) likely play a significant role. While it takes several decades for some lichenised fungi to reach their optimal size (Sancho et al. 2007), the short lifespan of leaves forces organisms that grow on them to have much shorter generation times, likely increasing diversification rates.

## ﻿Conclusion

This study represents a substantial advance in our understanding of the *Gomphillaceae*, providing the first comprehensive worldwide molecular phylogeny covering major tropical forest biomes. It highlights outstanding amounts of undescribed species and reveals more restricted distributions than previously assumed. It provides the basis for future studies on the taxonomy and phylogeography of this family and a framework to unveil more of their cryptic and underestimated diversity. Challenges remain in fully elucidating the global biogeography and species richness in *Gomphillaceae*, particularly in acquiring data from the gap regions identified in our study and in obtaining a greater number of loci per specimen. Developing effective protocols to facilitate the acquisition of molecular data would significantly advance our understanding of the evolutionary history of this family and help test hypotheses regarding cryptic species. Moreover, herbaria represent a highly promising avenue for addressing geographical gaps and resolving taxonomic issues, as they house extensive collections of foliicolous lichens, alongside vascular plant specimens. Overcoming the constraints posed by the small size of these lichens will enable future studies to explore population genetics in order to understand the direction of the long-distance dispersal events and colonisation patterns that shape the biogeography of the *Gomphillaceae*. This could involve more intensive sampling focused on specific clades or species complexes and the integration of additional molecular markers (e.g. ITS) or advanced techniques (e.g. involving NGS data).

## ﻿Additional information

### Adherence to national and international regulations

Specimen collection was conducted under the following permits: nos. 2022-03 and 2023-17 issued by the Guadeloupe National Park (PNG) and an additional permit AB/2.25 from the National Forestry Office for areas outside PNG. In New Caledonia, collection permits nos. 395-2022/ARR/DDDT and 609011-18/2022/JJC were used for the North and South Provinces of New Caledonia, respectively. For Réunion, Madagascar and Mayotte, the collection permits were Dl R-1-2022-220 issued by the Réunion National Park, 170/14/MEF/SG/DGF/DCB.SAP/SCB, 273/19/MEDD/SG/DGEF/DGRNE, 271/22/MEDD/SG/DGGE/DAPRNE/SCBE.Re and 317/22/MEDD/SG/DGGE/DAPRNE/SCBE.Re for Madagascar and CL/CK/17017 issued by the Conservatoire du littoral, 118/DEAL/SEPR/2017 issued by the Direction de l’Environnement, de l’Aménagement et du logement and a collecting permission issued by the Office National des Forêts through the courtesy of Laurent Mercy and Agnès Thongo for Mayotte. In Peru, permits 001-2021-SERNANP-PNRA, 002-2022-SERNANP-JEF, 003-2022-SERNANP-JEF, 003666-SERFOR and 003757-SERFOR within the project “LOT01 Amazon” and 001-2023-SERNANP-PNYCh and 003873-SERFOR within the project “LOT02 Andes” were obtained. In Colombia, permit 003639 was obtained within the project “LOT03 Amazon-Andes”. For Taiwan, permits 1121100335, 1122104050 and 1122260474 were issued by the Ministry of Agriculture and permit 1121010232 was issued by the Taroko National Park. All newly-sequenced specimens from Brazil were collected under permits which are kept in the Brazilian Herbaria CGMS or ISE.

### Author ORCIDs

Elise Lebreton https://orcid.org/0000-0002-4628-6521

Damien Ertz https://orcid.org/0000-0001-8746-3187

Robert Lücking https://orcid.org/0000-0002-3431-4636

Andre Aptroot https://orcid.org/0000-0001-7949-2594

Fabian Carriconde https://orcid.org/0000-0003-3987-7662

Claudine Ah-Peng https://orcid.org/0009-0008-9208-514X

Jen-Pan Huang https://orcid.org/0000-0002-9329-8867

Ko-Hsuan Chen https://orcid.org/0000-0001-9099-9054

Pierre-Louis Stenger https://orcid.org/0000-0001-9302-8085

Marcela Eugenia da Silva Cáceres https://orcid.org/0000-0002-5612-1309

Pieter van den Boom https://orcid.org/0000-0002-1929-2088

Emmanuël Sérusiaux https://orcid.org/0000-0002-0456-0131

Nicolas Magain https://orcid.org/0000-0001-5409-9518

### Data availability

The DNA sequences generated in this study were deposited at the NCBI (GenBank) https://www.ncbi.nlm.nih.gov/genbank/.

## Supplementary materials

Supplementary material 1 GenBank accession numbers and voucher details for the taxa included in this analysis, with newly-generated sequences highlighted in bold Data type xlsx https://binary.pensoft.net/file/1253291This dataset is made available under the Open Database License (http://opendatacommons.org/licenses/odbl/1.0/). The Open Database License (ODbL) is a license agreement intended to allow users to freely share, modify, and use this Dataset while maintaining this same freedom for others, provided that the original source and author(s) are credited.Elise Lebreton, Damien Ertz, Robert Lücking, André Aptroot, Fabian Carriconde, Claudine Ah-Peng, Jen-Pan Huang, Ko-Hsuan Chen, Pierre-Louis Stenger, Marcela Eugenia da Silva Cáceres, Pieter van den Boom, Emmanuël Sérusiaux, Nicolas Magain

Supplementary material 2 Species delimitation results and geographical distribution for all specimens studied Data type xlsx https://binary.pensoft.net/file/1253292This dataset is made available under the Open Database License (http://opendatacommons.org/licenses/odbl/1.0/). The Open Database License (ODbL) is a license agreement intended to allow users to freely share, modify, and use this Dataset while maintaining this same freedom for others, provided that the original source and author(s) are credited.Elise Lebreton, Damien Ertz, Robert Lücking, André Aptroot, Fabian Carriconde, Claudine Ah-Peng, Jen-Pan Huang, Ko-Hsuan Chen, Pierre-Louis Stenger, Marcela Eugenia da Silva Cáceres, Pieter van den Boom, Emmanuël Sérusiaux, Nicolas Magain

Supplementary material 3 Sorensen index for the 32 administrative territories studied Data type xlsx https://binary.pensoft.net/file/1253293This dataset is made available under the Open Database License (http://opendatacommons.org/licenses/odbl/1.0/). The Open Database License (ODbL) is a license agreement intended to allow users to freely share, modify, and use this Dataset while maintaining this same freedom for others, provided that the original source and author(s) are credited.Elise Lebreton, Damien Ertz, Robert Lücking, André Aptroot, Fabian Carriconde, Claudine Ah-Peng, Jen-Pan Huang, Ko-Hsuan Chen, Pierre-Louis Stenger, Marcela Eugenia da Silva Cáceres, Pieter van den Boom, Emmanuël Sérusiaux, Nicolas Magain

Supplementary material 4 Estimated ancestral area probabilities for node A1 and B1 Data type xlsx https://binary.pensoft.net/file/1253294This dataset is made available under the Open Database License (http://opendatacommons.org/licenses/odbl/1.0/). The Open Database License (ODbL) is a license agreement intended to allow users to freely share, modify, and use this Dataset while maintaining this same freedom for others, provided that the original source and author(s) are credited.Elise Lebreton, Damien Ertz, Robert Lücking, André Aptroot, Fabian Carriconde, Claudine Ah-Peng, Jen-Pan Huang, Ko-Hsuan Chen, Pierre-Louis Stenger, Marcela Eugenia da Silva Cáceres, Pieter van den Boom, Emmanuël Sérusiaux, Nicolas Magain

Supplementary material 5 Phylogenetic tree on the complete dataset inferred by Maximum Likelihood analyses of the mtSSU, nuLSU and RPB1 regions Data type pdf https://binary.pensoft.net/file/1253295This dataset is made available under the Open Database License (http://opendatacommons.org/licenses/odbl/1.0/). The Open Database License (ODbL) is a license agreement intended to allow users to freely share, modify, and use this Dataset while maintaining this same freedom for others, provided that the original source and author(s) are credited.Elise Lebreton, Damien Ertz, Robert Lücking, André Aptroot, Fabian Carriconde, Claudine Ah-Peng, Jen-Pan Huang, Ko-Hsuan Chen, Pierre-Louis Stenger, Marcela Eugenia da Silva Cáceres, Pieter van den Boom, Emmanuël Sérusiaux, Nicolas Magain

Supplementary material 6 Geographical origin of major Gomphillaceae collections Data type pdf https://binary.pensoft.net/file/1253296This dataset is made available under the Open Database License (http://opendatacommons.org/licenses/odbl/1.0/). The Open Database License (ODbL) is a license agreement intended to allow users to freely share, modify, and use this Dataset while maintaining this same freedom for others, provided that the original source and author(s) are credited.Elise Lebreton, Damien Ertz, Robert Lücking, André Aptroot, Fabian Carriconde, Claudine Ah-Peng, Jen-Pan Huang, Ko-Hsuan Chen, Pierre-Louis Stenger, Marcela Eugenia da Silva Cáceres, Pieter van den Boom, Emmanuël Sérusiaux, Nicolas Magain

Supplementary material 7 Phylogenetic tree on the subset 1 inferred by Maximum Likelihood analyses of the mtSSU, nuLSU and RPB1 regions Data type pdf https://binary.pensoft.net/file/1253297This dataset is made available under the Open Database License (http://opendatacommons.org/licenses/odbl/1.0/). The Open Database License (ODbL) is a license agreement intended to allow users to freely share, modify, and use this Dataset while maintaining this same freedom for others, provided that the original source and author(s) are credited.Elise Lebreton, Damien Ertz, Robert Lücking, André Aptroot, Fabian Carriconde, Claudine Ah-Peng, Jen-Pan Huang, Ko-Hsuan Chen, Pierre-Louis Stenger, Marcela Eugenia da Silva Cáceres, Pieter van den Boom, Emmanuël Sérusiaux, Nicolas Magain

Supplementary material 8 Phylogenetic tree on the subset 1 inferred by Maximum Likelihood of the mtSSU, nuLSU and RPB1 regions Data type pdf https://binary.pensoft.net/file/1253298This dataset is made available under the Open Database License (http://opendatacommons.org/licenses/odbl/1.0/). The Open Database License (ODbL) is a license agreement intended to allow users to freely share, modify, and use this Dataset while maintaining this same freedom for others, provided that the original source and author(s) are credited.Elise Lebreton, Damien Ertz, Robert Lücking, André Aptroot, Fabian Carriconde, Claudine Ah-Peng, Jen-Pan Huang, Ko-Hsuan Chen, Pierre-Louis Stenger, Marcela Eugenia da Silva Cáceres, Pieter van den Boom, Emmanuël Sérusiaux, Nicolas Magain

Supplementary material 9 Phylogenetic tree on the subset 2 inferred by Bayesian analyses of the mtSSU, nuLSU and RPB1 regions Data type pdf https://binary.pensoft.net/file/1253299This dataset is made available under the Open Database License (http://opendatacommons.org/licenses/odbl/1.0/). The Open Database License (ODbL) is a license agreement intended to allow users to freely share, modify, and use this Dataset while maintaining this same freedom for others, provided that the original source and author(s) are credited.Elise Lebreton, Damien Ertz, Robert Lücking, André Aptroot, Fabian Carriconde, Claudine Ah-Peng, Jen-Pan Huang, Ko-Hsuan Chen, Pierre-Louis Stenger, Marcela Eugenia da Silva Cáceres, Pieter van den Boom, Emmanuël Sérusiaux, Nicolas Magain

Supplementary material 10 Graph showing the relationship between the number of specimens and the number of species Data type tiff https://binary.pensoft.net/file/1253300This dataset is made available under the Open Database License (http://opendatacommons.org/licenses/odbl/1.0/). The Open Database License (ODbL) is a license agreement intended to allow users to freely share, modify, and use this Dataset while maintaining this same freedom for others, provided that the original source and author(s) are credited.Elise Lebreton, Damien Ertz, Robert Lücking, André Aptroot, Fabian Carriconde, Claudine Ah-Peng, Jen-Pan Huang, Ko-Hsuan Chen, Pierre-Louis Stenger, Marcela Eugenia da Silva Cáceres, Pieter van den Boom, Emmanuël Sérusiaux, Nicolas Magain
