# Supplementary figures and images for: ﻿Global phylogeny of the family Gomphillaceae (Ascomycota, Graphidales) sheds light on the origin, diversification and endemism in foliicolous lineages
Source: IMA Fungus. 2025 Feb 17;16:e144194. doi: 10.3897/imafungus.16.144194 (PMC11882023; doi:10.3897/imafungus.16.144194)

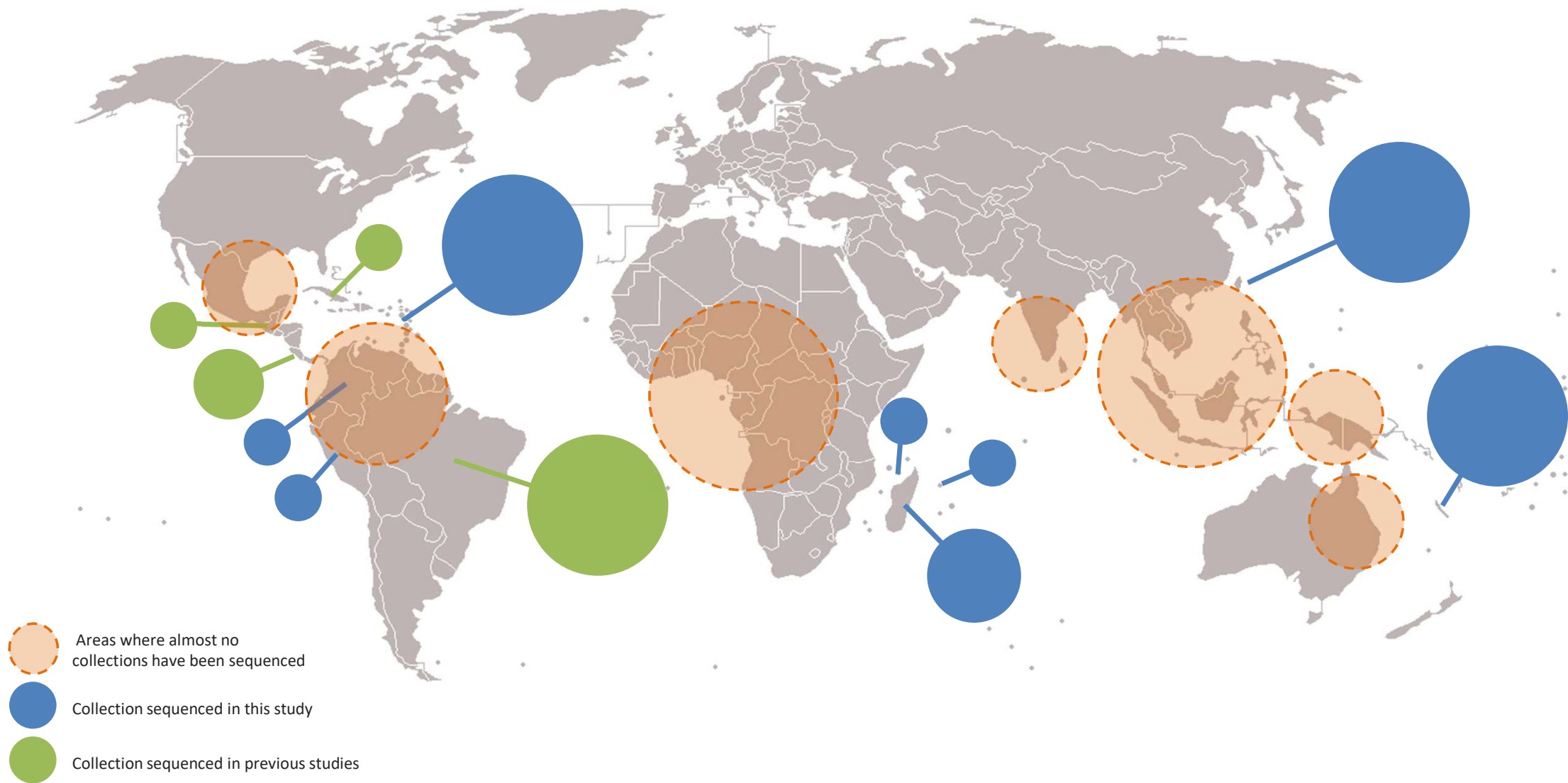

Supplement: Supplementary material 6 — Geographical origin of major Gomphillaceae collections [file imafungus-16-e144194-s006.pdf]

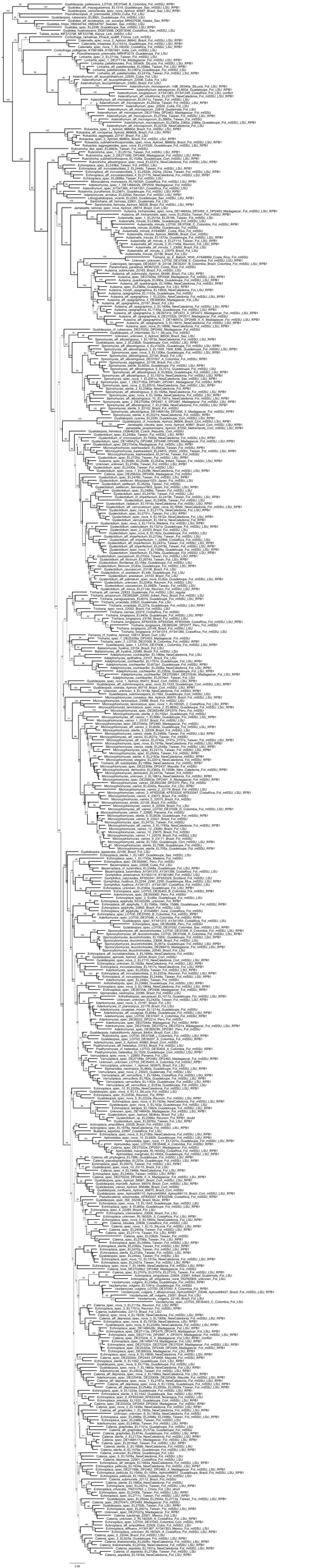

Supplement: Supplementary material 7 — Phylogenetic tree on the subset 1 inferred by Maximum Likelihood analyses of the mtSSU, nuLSU and RPB1 regions [file imafungus-16-e144194-s007.pdf]

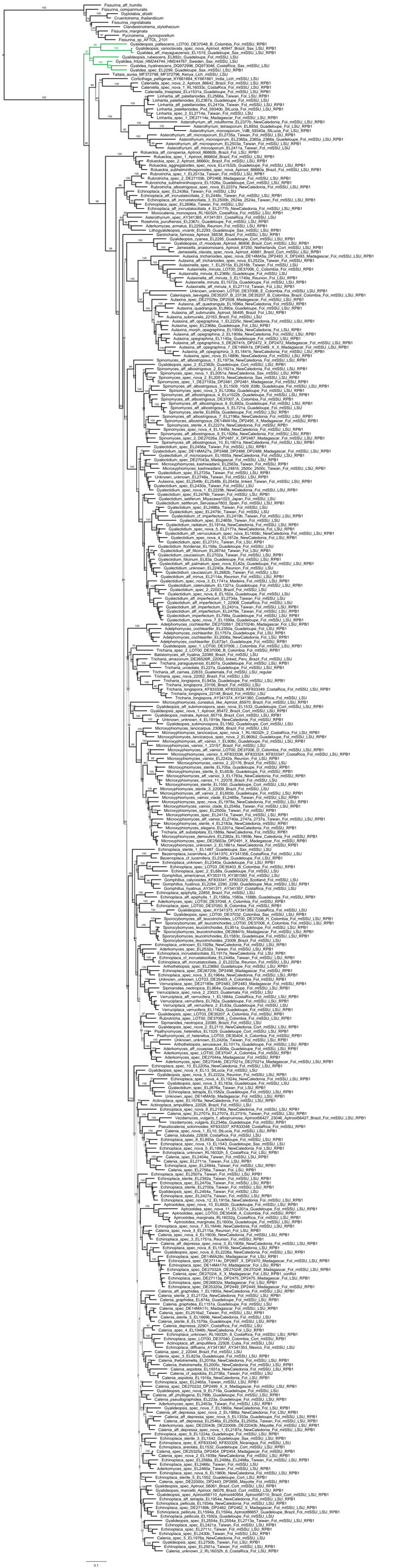

Supplement: Supplementary material 8 — Phylogenetic tree on the subset 1 inferred by Maximum Likelihood of the mtSSU, nuLSU and RPB1 regions [file imafungus-16-e144194-s008.pdf]

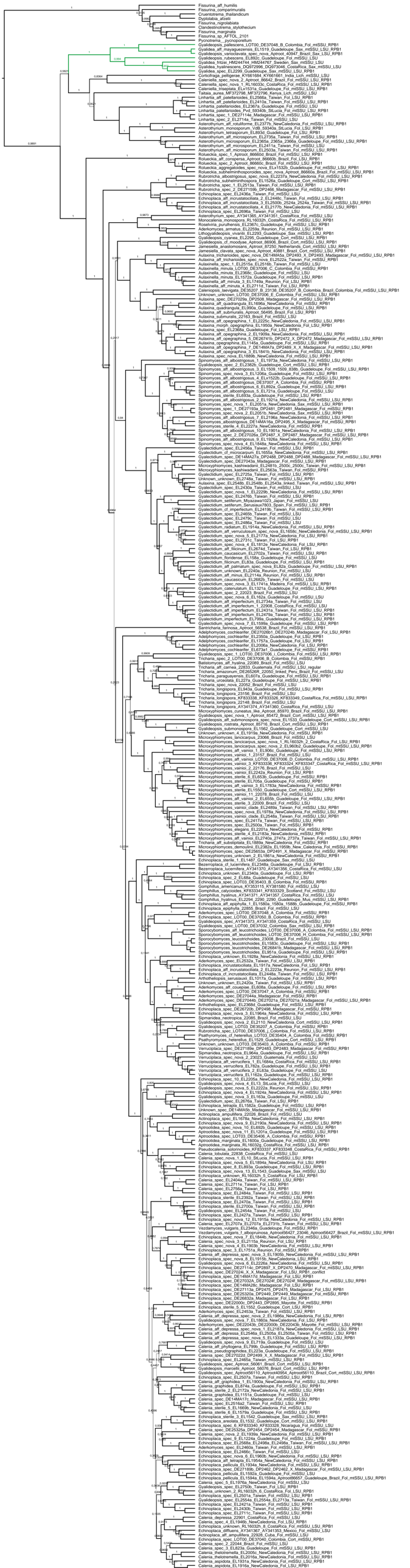

Supplement: Supplementary material 9 — Phylogenetic tree on the subset 2 inferred by Bayesian analyses of the mtSSU, nuLSU and RPB1 regions [file imafungus-16-e144194-s009.pdf]
