# Supplementary material for: ﻿Global phylogeny of the family Gomphillaceae (Ascomycota, Graphidales) sheds light on the origin, diversification and endemism in foliicolous lineages
Source: IMA Fungus. 2025 Feb 17;16:e144194. doi: 10.3897/imafungus.16.144194 (PMC11882023; doi:10.3897/imafungus.16.144194)
Supplement: Supplementary material 5 — Phylogenetic tree on the complete dataset inferred by Maximum Likelihood analyses of the mtSSU, nuLSU and RPB1 regions [file imafungus-16-e144194-s005.pdf]

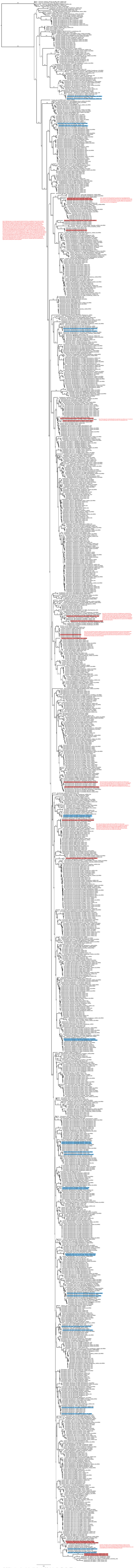

Fig. S1A. Phylogenetic tree on the complete dataset inferred by maximum likelihood analyses of the mtSSU, nuLSU and RPB1 region.
Specimens highlighted in red indicate the placement of the 18 specimens prior to concatenation into 9 specimens for species delimitation. Specimens marked in blue represent those concatenated after species delimitation, forming subset 1 used for BioGeoBEARS analyses.

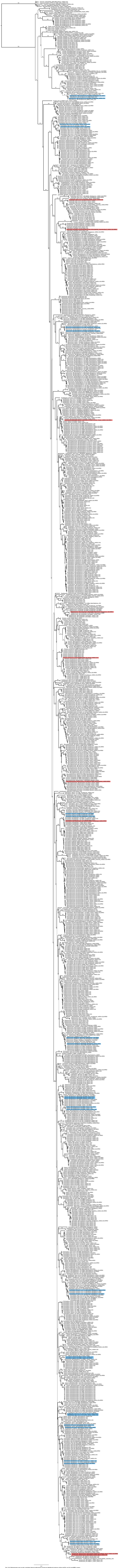

Fig. S1A. Phylogenetic tree on the complete dataset inferred by maximum likelihood analyses of the mtSSU, nuLSU and RPB1 region.
Specimens highlighted in red indicate the placement of **the 9 concatened specimens** for species delimitation. Specimens marked in blue represent those concatenated after species delimitation, forming subset 1 used for BioGeoBEARS analyses.
